# Evaluating the impact of pharmacists in outpatient mental health settings: a systematic review

**DOI:** 10.1007/s11096-026-02135-x

**Published:** 2026-04-15

**Authors:** Muireann Vaughan, Maria Donovan, Stephen Byrne, Fulvio Bedani, Sinéad O’Brien, Ciaran Halleran, Laura J. Sahm

**Affiliations:** 1 Pharmaceutical Care Research Group, School of Pharmacy, University College Cork, Cork, Ireland; 2 Pharmacy Department, Mercy University Hospital, Grenville Place, Cork, Ireland; 3 North Lee Mental Health Services, Health Service Executive, Cork, Ireland; 4 Cork Mental Health Services, Health Service Executive, Cork, Ireland

**Keywords:** Mental health services, Outpatient psychiatric clinical pharmacy, Pharmacist-led intervention, Psychiatric outpatient clinics

## Abstract

**Introduction:**

Mental disorders are estimated to affect one in seven people globally and are often linked to premature mortality. Psychotropic medicines play an important role in the management of many mental health conditions particularly where symptoms are moderate to severe, persistent, or complex. Pharmacists are increasingly recognised as key members of multidisciplinary mental health teams, with their role focusing on safe and effective use of psychotropic medicines. Mental healthcare is increasingly delivered in outpatient settings, however, the impact of pharmacists on these services remains underexplored.

**Aim:**

To systematically review and evaluate the impact of pharmacist-led or pharmacist-involved interventions specifically within outpatient mental health settings globally, across the full spectrum of mental disorders.

**Method:**

A systematic search of five electronic databases (MEDLINE, Embase, CINAHL, CENTRAL, and PsycINFO) was conducted from inception to October 2025. Grey literature sources including ClinicalTrials.gov and Google Scholar were also searched. Inclusion criteria were: (i) evaluation of a pharmacist-led or pharmacist-involved intervention with a measurable outcome; (ii) conducted in an outpatient mental health setting involving adults with a diagnosed mental disorder classified according to ICD (up to and including ICD-11) or DSM criteria (up to and including DSM-5), excluding dementia; and (iii) inclusion of a comparator or control group. Study screening, data extraction, and quality appraisal, were performed independently by two reviewers. Due to heterogeneity in study design and outcomes, a narrative synthesis was conducted.

**Results:**

Twenty-three studies met the inclusion criteria: 11 randomised controlled trials and 12 non-randomised studies. Pharmacist interventions varied, but most commonly included medication reviews, psychoeducation, adherence support and monitoring for side effects or drug interactions. Outcomes were grouped into seven domains: patient-reported, symptom-related, treatment-related, medication adherence, cost, monitoring and hospitalisations. Pharmacist impact was associated with improvements across multiple domains, particularly in medication adherence, treatment-related and symptom-related outcomes. While outcome measures varied between studies, a majority (82.6%) reported positive trends in at least one domain, favouring pharmacist-led or pharmacist-involved care over usual care.

**Conclusion:**

Pharmacist-led interventions in outpatient mental health settings are associated with positive clinical and healthcare-related outcomes across a range of mental disorders globally. These findings support the integration of pharmacists into multidisciplinary outpatient mental health teams and highlight the need for more high-quality, standardised research to inform service development and policy.

**Supplementary Information:**

The online version contains supplementary material available at 10.1007/s11096-026-02135-x.

## Impact statements

- This review highlights the evidence of clinical value of integrating pharmacists into outpatient mental health teams, demonstrating their positive contribution to symptom control, medication adherence, and treatment optimisation.
- Findings support the expansion of pharmacist roles in community mental health settings, aligning to a global shifts towards inclusion of pharmacists in outpatient and preventative care models.
- The review provides a foundation for policymaking and the design of future pharmacist-led mental health interventions and recommends standardised outcome reporting in this under-researched setting.

## Introduction

Mental disorders (MD) encompass a broad spectrum of conditions that affect mood, cognition, and behaviour, collectively posing a substantial global burden in terms of disability, morbidity and premature mortality [1, 2]. In 2019, approximately 418 million disability-adjusted life years (DALYs) were attributable to MD, accounting for about 5% of global DALYs [3]. Across the diagnostic spectrum, people with serious mental illness (SMI) lose on average 14.7 years of life, compared to the general population, largely due to physical comorbidities and health inequalities [4]. Individuals with MD experience markedly higher rates of cardiovascular disease, diabetes, obesity, and respiratory illness, which contribute to increased mortality [5, 6]. Pharmacists, as highly accessible and trusted healthcare professionals, are well-positioned to support the delivery of outpatient mental health (MH) care [7]. While non-pharmacological interventions are central to care for some conditions, pharmacological therapy remains an important component of treatment for many individuals with MD, particularly those with moderate to severe or complex/enduring symptoms [8, 9]. In this context, integrating pharmacists within the multidisciplinary team (MDT) represents a logical approach to optimising medication use and supporting holistic, patient-centred care.

Over the past two decades, MH service delivery has shifted significantly from institutional and inpatient-based care towards outpatient models delivered across community, primary care e.g. General Practice, and hospital outpatient settings [10]. This evolution reflects a growing recognition that MD are often chronic, requiring long-term management [11]. Outpatient care supports continuity, accessibility and early intervention, and is now regarded as a preferred standard of care for many MH conditions, particularly where symptoms can be safely managed outside hospital environments [12, 13]. International policy frameworks, including the World Health Organisation’s (WHO) Comprehensive Mental Health Action Plan 2013–2030, recommends the expansion and strengthening of outpatient MH services embedded broader health systems [14].

Internationally, clinical pharmacy services in MH care are increasingly recognised as a component of MDT-based practice, although the scope, maturity, and implementation of these services vary widely across health systems. A recent commentary by the European Society of Clinical Pharmacy (ESCP) Special Interest Group (SIG) on Mental Health highlights the growing role of clinical pharmacists in mental health care across Europe, while also identifying persistent challenges related to service integration, funding, and evaluation [15]. Pharmacists’ roles in MH care now extend beyond traditional dispensing functions to encompass clinical activities e.g. adherence support, cardiometabolic and physical health monitoring, and collaborative decision-making within MDTs [16, 17]. In some European countries, including for example; the United Kingdom (UK) and Slovenia, pharmacists are already embedded within outpatient and ambulatory mental health services, with roles extending, in certain settings, to pharmacist independent prescribing and structured medication review across care interfaces [18–20]. Recent qualitative evidence has highlighted the perceived benefits of pharmacist independent prescribing for individuals with mental illness in community and outpatient settings, including improved access to care and enhanced continuity of medication management [19]. These advances demonstrate the feasibility and acceptability of pharmacist integration within outpatient mental health services, while underscoring the importance of robust evaluation to inform service design and policy.

Previous reviews have reported positive impacts of pharmacist involvement across mental health settings, including improvements in medication adherence, optimisation of pharmacotherapy, patient satisfaction, and medication safety [21–25]. However, existing syntheses have largely focused solely on severe and persistent mental illness, community pharmacy-based interventions, diagnosis-specific interventions or mixed healthcare settings, limiting conclusions regarding the specific impact of pharmacist-led, or pharmacist-involved, interventions within outpatient mental health settings.

## Aim

To identify, synthesise, and evaluate evidence of the impact of pharmacist-led, or pharmacist-involved, interventions within outpatient mental health settings.

## Methods

This review was guided by the Cochrane Handbook for Systematic Reviews of Interventions and is reported in accordance with the PRISMA (Preferred Reporting Items for Systematic Reviews and Meta-Analysis) guideline [26, 27]. The protocol was registered in the PROSPERO register, CRD42025629212 [28].

### Search strategy and selection criteria

Potentially relevant studies published from inception to 22nd-October-2025 were identified by searching the following databases: MEDLINE(Ebsco), Embase(Elsevier), PsycINFO(Ebsco), CINAHL(Ebsco), CENTRAL (Cochrane Library). In addition, reference lists of relevant reviews were screened for potentially relevant individual studies. A grey literature search was conducted by screening records on ClinicalTrials.gov and reviewing the first 100 results returned from a Google Scholar search using the primary keywords of the review.

The following search terms or subject headings (and related concepts as title and abstract searches, depending on the database) were used.

*Concept 1*

Pharmacist OR clinical pharmacist OR pharmacy.

AND

*Concept 2*

Psychiatry OR mental health service OR, persons with mental disorders OR substance abuse OR eating disorders OR enuresis OR behaviour addictive OR mental disease NOT dementia;

AND

*Concept 3*

Outpatient OR outpatient service OR community mental health service OR ambulatory care OR outpatient clinic.

A full search strategy is available as Online Resource 1.

### Study selection and data extraction

Search results were downloaded into Covidence (Covidence Systematic Review Software), duplicates were removed manually and automatically. Two reviewers independently screened all abstracts and full-texts for potentially eligible studies. Any disagreements were resolved by a third independent assessor.

The following data were extracted where possible as per the terminology used in the study: author(s), year, country, study aim, setting, sample size, population, study design, intervention(s), outcomes related to impact of pharmacists’ intervention(s), pharmacists’ role(s).

### Inclusion/exclusion criteria

The inclusion criteria were formulated using the PICOS (Participant, Intervention, Comparison, Outcome and Study design) [26]. Studies were included if they met the following criteria:

*Participants:* The study population involved adults aged 18 years and over, diagnosed with a MD classified according to the International Classification of Diseases (ICD; up to and including the 11th Revision, including ICD-10) or the Diagnostic and Statistical Manual of Mental Disorders (DSM; up to and including DSM-5), excluding dementia, who have accessed pharmacist services within their outpatient MH care.

*Intervention:* Any activities delivered by a pharmacist/pharmacy student/pharmacy support staff within an outpatient MH setting that had a measurable impact on service users. These activities may have included, but were not limited to, medication counselling, treatment recommendations or clinical monitoring, provided they were associated with a quantifiable outcome.

*Comparator and Outcome:* Outcome domains were not defined *a-priori*. Outcomes were eligible for inclusion if they were attributable to (i) pharmacist-led, or pharmacist-involved, interventions, (ii) demonstrated a quantifiable and measurable impact, and (iii) were assessed against a comparator other than baseline. All reported outcomes were extracted and subsequently grouped inductively into seven outcome domains to support narrative synthesis across heterogeneous study designs. A detailed summary of the outcome measures grouped within each domain is provided in Online Resource 2.

### Outcome domains

1. Patient-reported
2. Symptom-related
3. Treatment-related
4. Medication adherence
5. Cost
6. Monitoring
7. Hospitalisations

For studies with a mixed population of inpatient and outpatient settings, studies were included if the impact(s) of pharmacists’ involvement in outpatient settings could be extracted.

*Study Design:* All primary research was considered for inclusion, regardless of study design or language of publication.

The following types of studies were excluded:

- Studies relating only to dementia patients.
- Non-primary research publications, such as conference abstracts, case reports, editorials and letters.

*Setting and Context:* Eligible studies were conducted in outpatient mental health settings, defined as non-inpatient clinical environments where care is delivered through hospital outpatient departments or specialist outpatient mental health clinics. This definition reflects the structure of outpatient psychiatry services within the Irish healthcare system. Studies conducted in nursing homes, primary-care, emergency departments, acute ambulatory care settings for psychosis presentations, community pharmacies, or residential care facilities for individuals with intellectual disabilities were excluded.

### Risk of bias (ROB)

The ROB was assessed using the Cochrane ROB tool for randomised trials (RoB 2) and Risk Of Bias In Non-randomised Studies of Interventions Version 2 (ROBINS-I V2) [29, 30]. Two reviewers independently conducted the ROB assessments. Randomised Controlled Trials (RCTs) were evaluated across five RoB 2 domains: randomisation process, deviations from intended interventions, missing outcome data, measurement of the outcome and selection of reported result. For non-randomised studies, the ROBINS-I V2 tool was used to assess bias across seven domains: confounding, selection of participants, classification of the intervention, deviation from intended intervention(s), missing data, measurement of outcome(s), and selection of reported results. Each ROB item was rated as either low, some concerns/moderate or critical/high ROB. No studies were excluded based on this assessment.

## Results

**Fig. 1:**
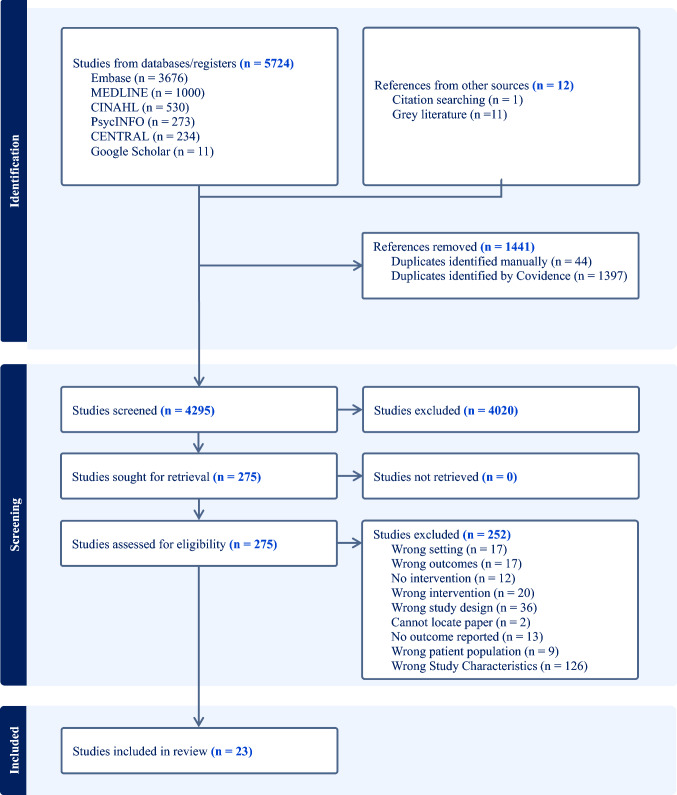
PRISMA flow diagram showing process of study selection for inclusion in systematic review

The literature search yielded 5,736 potentially relevant citations. After 1,441 duplicates were removed, 4295 citations were title and abstract screened, with 275 full-text articles assessed for eligibility. Following full-text screening, 23 studies were deemed eligible for inclusion in the review. Substantial heterogeneity in study design and outcome measures limited the feasibility of meta-analysis, therefore, a narrative synthesis approach was applied. Figure 1 depicts the PRISMA flowchart of the article selection process.

### Study characteristics

Twenty-three studies, spanning ten countries, with the largest proportion (n = 10) conducted in the USA [31–40] and comprising eleven RCTs [40–50], four pre–post intervention studies [31, 34, 38, 51], five retrospective cohort studies [32, 33, 36, 37, 52], two prospective cohort studies [35, 39], and one prospective longitudinal crossover study [53], were included. Fifteen studies were conducted in outpatient clinics [32–40, 45, 46, 49, 51–53] with one study conducted between an outpatient clinic and a home care setting [31]. Seven studies were carried out in the outpatient department of a hospital [41–44, 47, 48, 50]. Six studies did not specify participants’ diagnoses [31, 32, 34, 37, 39, 40], and one study reported mixed mental disorder diagnoses [33]. The sample size of included studies ranged from 10 to 443 participants. Two studies reported the number of clinic visits rather than individual participants [34, 38]. Across the remaining 21 studies, a total of 2,256 participants were included across various outpatient settings. Study duration was reported in all studies, ranging from 6 weeks to 10 years.

### Outcome measures of impact

Outcomes were grouped into seven domains: (i) symptom-related outcomes, (ii) treatment-related outcomes, (iii) medication adherence, (iv) patient-reported outcome measures (PROMs), (v) monitoring, (vi) hospitalisations and (vii) cost. Table 1 provides the results of each study by outcome domain and an overview of the study characteristics.

**Table 1:** Overview of included studies

Year, First Author, Country	Study name	Study design, setting	Diagnoses, sample size (n)	Key findings
2025, Mishra, India	Randomized controlled trial to assess medication adherence and health-related quality of life (QoL) through a collaborative pharmacist-psychiatrist approach to patient education in patients with depression in India	Randomised Controlled Trial (RCT), Outpatient Department (OPD)	Depression, n = 75	***Medication Adherence*** The collaborative care group showed a statistically significant improvement in medication adherence as measured by the Medication Adherence Rating Scale, with a mean increase of 1.67 ± 0.25 (*p* < 0.001), compared with a mean increase of 0.69 ± 0.05 (*p* < 0.05) for the usual care group ***Symptom Related*** Health Related Quality of Life (HRQoL) scores, as measured by the World Health Organisation Quality of Life-Brief (WHOQOL-BREF) Scale, improved significantly more in the collaborative care group, with a mean increase of 28.01 ± 2.05 (*p* < 0.001), compared with a mean increase of 12.46 ± 0.26 (*p* < 0.05) for the usual care group
2024, Chang, USA	Rural ambulatory care pharmacists providing in-clinic and home visit services improve adherence to long-acting injectable (LAI) antipsychotics	Pre-post intervention study, Ambulatory care clinics and home care settings	n/r, n = 10	***Treatment related*** The percentage of days covered by LAI fills increased from an average of 26% to 67% of days covered (*p* = 0.06) ***Hospitalisations*** Total Emergency Department (ED) visits related to mental health episodes decreased from 11 to 2 visits (*p* = 0.03) ***Monitoring*** The percentage of patients that had laboratory monitoring completed in the previous year increased from 30 to 80%
2024, Olson, USA	A retrospective comparison of pharmacist and psychiatrist-led medication management clinics in an outpatient setting	Retrospective cohort study, Outpatient clinic	non- specific, n = 221	***Medication Adherence*** The pharmacist-led clinic had a significantly higher Medication Possession Ratio (MPR) (*p* < 0.00001) ***Hospitalisations*** ED visits and admissions per patient were similar between the pharmacist and psychiatrist groups (0.045 ± 0.248, 0.081 ± 0.307, respectively; *p* = 0.646) ***Monitoring*** The pharmacist-led clinic had a significantly higher rate of completed laboratory monitoring (*p* = 0.0015)
2023, Gregorian, USA	Design, implementation, and evaluation of a pharmacist-led outpatient benzodiazepine-tapering clinic	Retrospective cohort study, Benzodiazepine taper clinic	n/r, n = 159	***Treatment Related*** 90% of patients had at least some benzodiazepine tapering when enrolled in the clinic, compared to 41% among not enrolled in the clinic (*p* < 0.001). 27% of patients enrolled in the clinic were completely tapered off benzodiazepine in comparison to 4% of those not enrolled (*p* < 0.001)
2023, Vickery, USA	Increasing access to psychiatric care during the COVID-19 pandemic through mental health clinical pharmacy specialist services	Prospective cohort study, Outpatient clinic	n/r, n = 45	***Patient Reported*** Treatment Satisfaction scores were high in both groups. The most frequent score among all surveys was 4.8 on a 5-point scale, (*p* > 0.05) indicating no statistically significant differences between clinician types
2022, Spann, Australia	Pharmacists in clozapine clinics improving physical health monitoring	Retrospective cohort study, Outpatient clinic	Schizophrenia, n = 61	***Monitoring*** Pharmacist clinic had statistically higher rates of metabolic monitoring (glucose 48% vs 11%, *p* < 0.001; lipids 61% vs 7.1%, *p* < 0.001) and Electrocardiogram (ECG) monitoring (15% vs 0%, *p* < 0.001)
2021, Mailoux, USA	Development and implementation of a physician-pharmacist collaborative practice model (PPCM) for provision and management of buprenorphine/naloxone	Prospective cohort study, Outpatient clinic	OUD, n = 50	***Monitoring*** Collection of Urine Drug Samples was more frequent in PPCPM appointments, 98% versus 89% of psychiatrist-only appointments
2021, Mattle, USA	Evaluating outcomes of a clinical pharmacist medication management program in a multidisciplinary practice for outpatient buprenorphine treatment of opioid use disorder	Retrospective cohort study, Outpatient clinic	OUD, n = 150	***Medication Adherence*** Significantly more buprenorphine nonadherence at the physician-only practice (*p* < 0.01) ***Patient Reported*** Similar rates of engagement with counselling (*p* = 1) were observed in both groups
2021, Pohl, USA	Implementation of a community-based pharmacist-run attention deficit hyperactivity disorder clinic in a college health centre	Pre-post intervention study, Outpatient clinic	ADHD, 443 visits	***Monitoring*** Compared with psychiatrist-run appointments, pharmacist-run appointments were more adherent to monitoring blood pressure (11% vs. 77%, *p* < 0.001) and heart rate (6% vs.75%, *p* < 0.001)
2020, Salazar-Ospina, Colombia	Long-term impact of pharmacist intervention in patients with bipolar disorder: extended follow-up to the EMDADER-TAB study	RCT, Outpatient clinic	Bipolar 1 Disorder (BD-1) n = 92	***Hospitalisations*** One year after pharmacist intervention ceased, there were no significant differences between groups in psychiatric hospitalisations (*p* = 0.261). There were 14 emergency service consultations for the Intervention Group (IG) versus 24 for the Control Group (CG) (*p* = 0.212)
2018, Sathienluc-kana, Thailand	Anticholinergic discontinuation and cognitive functions in patients with schizophrenia: A pharmacist–physician collaboration in the outpatient department	RCT, OPD	Schizophrenia, n = 41	***Treatment Related*** Drug Related Problems were reduced by 85.19% in the pharmacist intervention group and by 9.76% in the usual care group ***Symptom Related*** Executive function perseverative errors within the pharmacist intervention group improved significantly from baseline (*p* = 0.003). Brief Psychiatry Rating Scale scores were significantly lower in the pharmacist intervention group than the usual care group (36.08 (± 3.62) to 24.69 (± 2.72) and 38.29 (± 6.38) to 36.35 (± 7.71), respectively; (*p* < 0.001)
2018, Lindell, USA	A pilot evaluating clinical pharmacy services in an ambulatory psychiatry setting	Retrospective cohort study, Outpatient clinic	Depression, Anxiety, Other n = 217	***Medication Adherence*** Patient self-reported adherence found a higher adherence rate in the intervention group (*p* < 0.0001) ***Symptom Related*** No significant difference was found in PHQ-9 (*p* = 0.87) or General Anxiety Disorder (GAD) (*p* = 0.75) scores between groups
2017, Mishra, India	Impact of pharmacist-psychiatrist collaborative patient education on medication adherence and quality of life of Bipolar Affective Disorder (BPAD) patients	RCT, OPD of Psychiatric Hospital	BPAD, n = 75	***Medication Adherence*** Improvement in medication adherence in the IG as measured by the Medication Adherence Rating Scale (MARS) was found to be 2.06 (± 0.15) (*p* < 0.001) ***Symptom Related*** Mean improvement in QOL, as measured by the WHO-BREF SCALE, of the test and control groups was found to be 13.8 (± 10.5) (*p* < 0.05)
2017, Mishra, India	Impact of pharmacist-led collaborative patient education on medication adherence and quality of life of schizophrenia patients in a tertiary care setting	RCT, OPD of Psychiatric Hospital	Schizophrenia, n = 26	***Medication Adherence*** Both groups showed an increase in medication adherence as measured by the MARS. The intervention group had a larger and more statistically significant increase (*p* = 0.003) ***Symptom Related*** Assessment of patient’s QOL, using the WHOQOL-BREF Scale between groups showed a mean(SD) improvement of 8.05(5.32) in QOL of the IG over the CG (*p* < 0.001)
2017, Salazar-Ospina, Colombia	Effectiveness of the Dader method for pharmaceutical care on patients with bipolar I disorder: Results from the EMDADER-TAB study	RCT, Outpatient clinic	BD-I, n = 92	***Hospitalisations*** hospitalisations and emergency service consultations was higher for the CG than for the IG (Hazard Ratio (HR) = 9.03, *p* = 0.042; HR = 3.38, *p* = 0.034, respectively)
2017, Singh, India	The impact of clinical pharmacist lead collaborative care on quality of life of the patients with bipolar disorder: A unicenter prospective, randomization study	RCT, OPD	BPAD, n = 286	***Symptom Related***nt improvement in quality of life, as measured by the WHO-BREF scale, was observed during the study period (*p* < 0.001)
2015, Aljumah, Saudi Arabia	Impact of pharmacist intervention using shared decision making on adherence and measurable depressed patient outcomes	RCT, OPD of Psychiatric Hospital	Major Depressive Disorder, n = 239	***Patient Reported*** Patient Satisfaction with treatment as measured by the Treatment Satisfaction Questionnaire for Medication was significantly higher in the IG (*p* < 0.0001) ***Medication Adherence*** Patients in the intervention group had significantly more favourable medication adherence as measured by the Morisky Medication Adherence Scale (MMAS) (*p* < 0.0001) ***Symptom Related*** The groups did not differ in severity of depression as measured by the Montgomery–Åsberg Depression Rating Scale (*p* = 0.971)
2014, Schneiderhan, USA	Twelve-Month Prospective Randomized Study of Pharmacists Utilizing Point-Of-Care Testing for Metabolic Syndrome and Related Conditions in Subjects Prescribed Antipsychotics	RCT, Outpatient clinics	n/r, n = 120	***Symptom related*** Between group differences in adjusted mean number of metabolic syndrome parameters at six months (*p* = 0.24) and 12 months (*p* = 0.99) were not statistically significant
2013, Alves, Brazil	Assessment of the Effectiveness of Pharmacotherapy Follow-up in Patients Treated for Depression	RCT, Outpatient clinic,	Depression n = 58	***Symptom Related*** A comparison of CG and IG showed a statistically significant difference between groups, with a median reduction in BDI score (Δ) of 2.5 points in the CG and 13.5 points in the IG (*p* = 0.0275)
2008, Al-Saffar, Kuwait	Depressed patients' preferences for education about medications by pharmacists in Kuwait	RCT, OPD of Psychiatric Hospital	Depression, n = 150	***Patient Reported*** Counselling was found to be significantly associated with a much higher recall of medicine name (Odds Ratio (OR) = 9.6, *p* = 0.01), how to manage missed doses (OR = 8.9, *p* = 0.007), and correct use of medication (OR = 31.3, P < 0.001). Leaflet use was less strongly associated than counselling and was statistically significant for recall regarding correct use of medication (OR = 8.4, *p* = 0.009)
2006, Fridman, Argentina	Pharmaceutical care in the follow-up of psychiatric outpatients	Prospective longitudinal non-randomised, crossover study, Outpatient clinic	Psychosis, n = 75	***Treatment Related*** Drug Related problems reduced significantly in both groups during the intervention period (Group A: 46.65% decrease, Group B: 52.63% decrease)
1993, Dorevich, Israel	Medication maintenance of chronic schizophrenic out-patients by a psychiatric clinical pharmacist: 10-year follow-up study	Pre-post intervention study, Outpatient clinic	Schizophrenia, n = 14	***Treatment Related*** No. of medication-related side-effects per patient was significantly less at the end of the study period (*p* = 0.0033) ***Hospitalisations*** No. of days of hospitalisation for psychiatric reasons reduced from 684 days in the 10-year pre study period to 102 days in the 10-year study period
1989, Lobeck, USA	The cost-effectiveness of a clinical pharmacy service in an outpatient mental health clinic	Pre-post intervention study, Outpatient clinic	n/r, 7396 clinic visit	***Cost*** Total Cost per Prescription (Drug + Personnel) decreased by 14.3% by the end of the intervention period. The Drug cost per prescription decreased by 34.6% across the same period

### Pharmacist roles

Medication-related counselling was the most frequently reported pharmacist role (n = 19), followed by treatment/medication recommendations (n = 11) and medication adherence assessment/counselling (n = 11). Pharmacist involvement included clinical assessment (n = 8) and lifestyle assessment/counselling (n = 6).​ Pharmacists also provided information to patients of medication interactions (n = 2), medication related monitoring (n = 5) and administration of long-acting injectable antipsychotics (n = 1). Online Resource 3 gives an overview of the pharmacist roles in each study.

### Risk of bias

#### RCTs

**Fig. 2:**
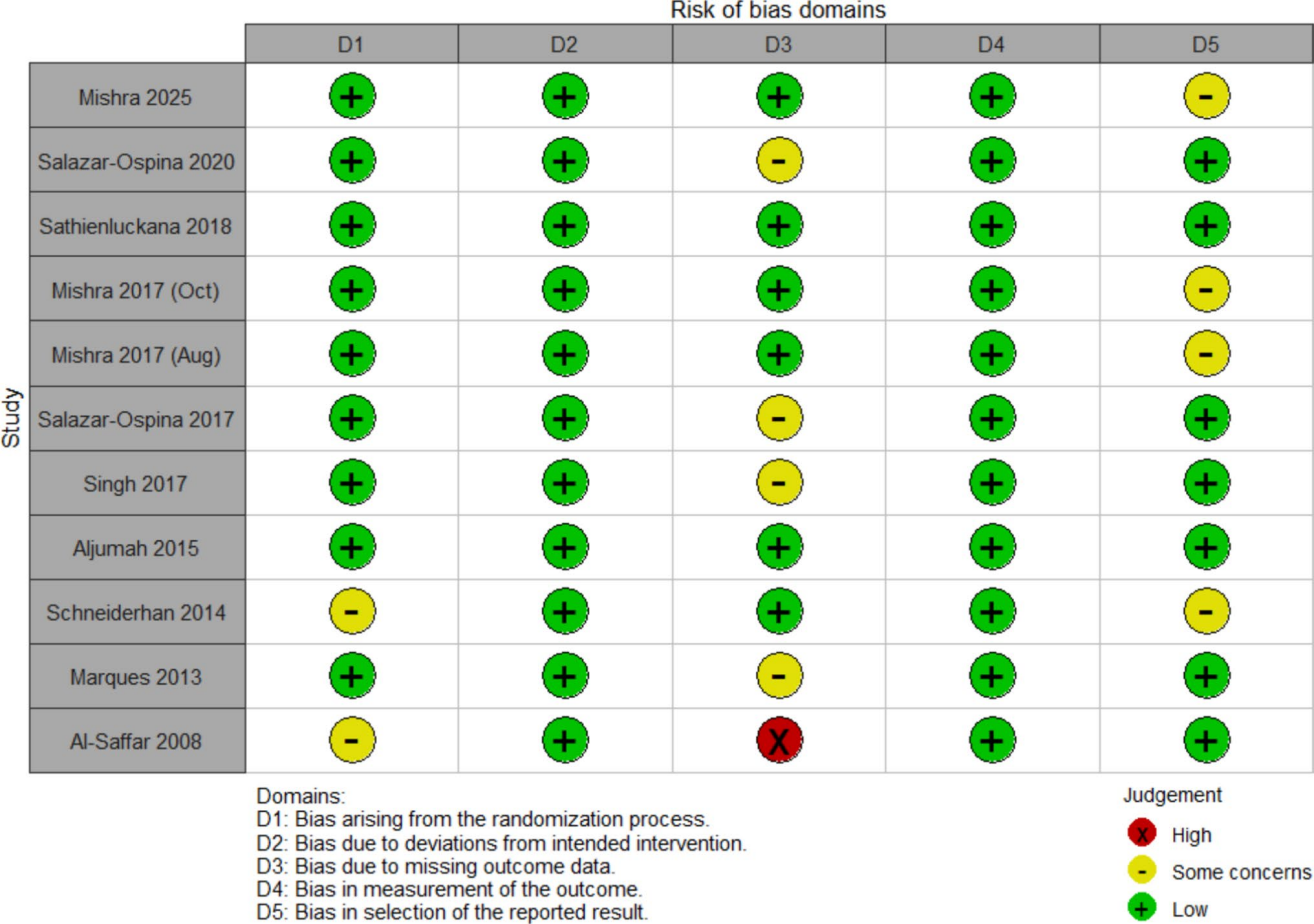
Risk of bias of randomised controlled trials using RoB 2 tool

ROB for the RCTs was assessed using the RoB 2 tool [29]. In Domain 1, nine studies were rated at low risk [42–50], while two studies [40, 41] were rated at moderate risk as group assignment was determined by the day of recruitment or block randomisation, not by truly random methods. In Domain 2, all eleven studies were judged at low risk. For Domain 3, six studies [40, 42–44, 47, 50] were assessed as low risk; four studies [45, 46, 48, 49] were rated at moderate risk; and one study [41] was judged to be at high risk due to a high loss to follow-up after the interventions. All eleven studies were rated as low risk in Domain 4. In Domain 5, seven studies [41, 42, 45–49] were assessed as low risk, while four studies [40, 43, 44, 50] were rated at moderate risk as the presence of a trial protocol/prespecified analysis plan was not mentioned in these studies (Fig. 2).

#### Non-RCT

**Fig. 3:**
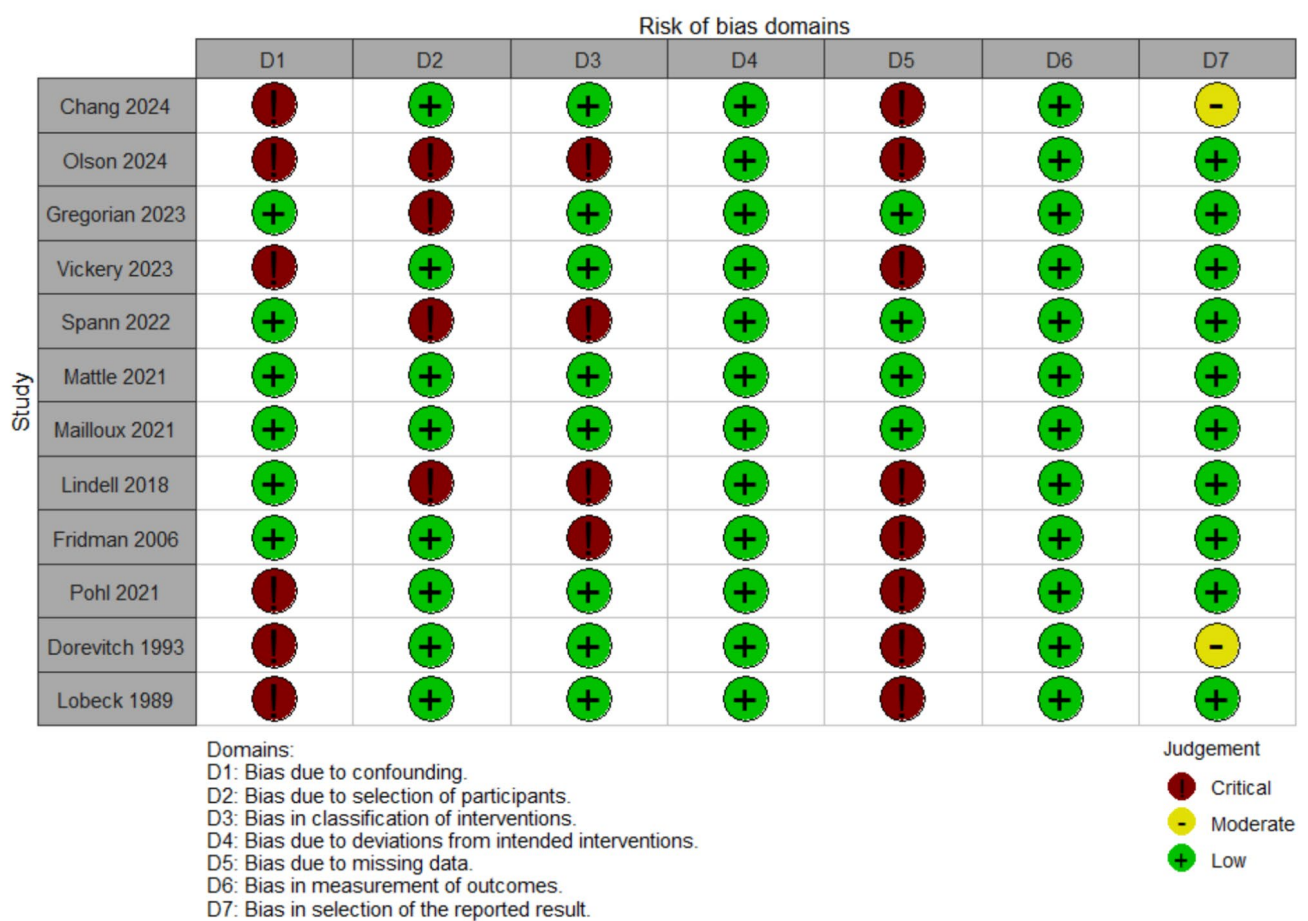
Risk of bias of nonrandomised controlled trials using ROBINS-I V2 tool

ROB for the 12 non-randomised studies was assessed using the ROBINS-I V2 tool [30]. The most frequent sources of bias were related to missing data, with eight studies [31, 33, 34, 37–39, 51, 53] judged as high risk**,** and confounding, with half of the studies (n = 6) [31, 34, 37–39, 51] rated as high risk. All studies were assessed as low risk for deviations from intended interventions and all but one study [31] were classed as low risk for selection of the reported result. A detailed breakdown by domain for each study is presented in Fig. 3.

## Discussion

This review provides a comprehensive overview of the outcomes associated with pharmacist involvement in outpatient MH services and highlights the measurable impact of pharmacist-led interventions across a range of MD. Overall, pharmacist involvement was associated with improved outcomes, particularly in the domains of treatment-related outcomes, medication adherence, and monitoring, where all studies[31–33, 35–38, 42–44, 47, 50–53] assessing these outcomes reported positive effects.

The value of pharmacists in MH care has been increasingly recognised with several studies highlighting their contribution to improved clinical outcomes, medication management, and interdisciplinary collaboration in psychiatric settings [17, 23, 50, 54–56]. Recent international literature further emphasises the expanding role of clinical pharmacy services within mental health care across Europe. A commentary by the ESCP SIG on Mental Health highlights both the progress made and the ongoing variability in the implementation of mental health clinical pharmacy services across European health systems, including outpatient and ambulatory settings [15]. In the UK, for example, The NHS Confederation/Centre for MH report of 2017 on the future of the MH workforce included a section on pharmacy referring to pharmacy as “*an untapped resource… for the huge challenge MH services have supporting people with medication*”[57]. In addition, pharmacists in UK outpatient and community mental health teams have increasingly adopted independent prescribing roles, further embedding pharmacy within specialist multidisciplinary services and expanding direct patient care responsibilities [18, 19]. Similarly, in Slovenia, structured clinical pharmacy services have been implemented within primary care settings, including medication review services for patients with mental disorders and interdisciplinary collaboration within psychiatric services, demonstrating the feasibility of pharmacist integration within European outpatient mental health contexts [20, 58].These developments demonstrate growing international recognition of the contribution pharmacists can make to outpatient mental health services and reinforce the importance of robust evaluation to inform service planning and sustainability.

All five studies that assessed treatment-related outcomes reported beneficial effects associated with pharmacist involvement. Two studies reported a reduction in the number of DRP following pharmacist involvement, suggesting a positive impact on medication safety and optimisation [47, 53]. These findings can also be found in non‑psychiatric settings, Al‑Taani et al*.* demonstrated that clinical pharmacists in internal medicine outpatient clinics in Jordan identified numerous DRP, and interventions to address them resulted in avoided costs and improved medication management [59]. Together, these results suggest that pharmacists may contribute to safer and more effective treatment and have the potential to influence broader clinical outcomes.

Nine studies included symptom-related outcomes, with six of these reporting clinical improvements [43, 44, 47–50]. Symptom measurement typically requires condition-specific, validated tools (e.g., PHQ-9, GAD-7, PANSS), which may not be consistently available or used in routine outpatient care [60–62]. The heterogeneity of psychiatric diagnoses and variability in follow-up periods across studies may further complicate the selection and timing of appropriate measures [63]. There is also inconsistency in the use of clinician-rated, versus patient-reported, instruments, which can affect comparability of results. Nonetheless, guidance from the American Psychiatric Association via the DSM-5-Text Revised supports the routine use of symptom rating scales at baseline and during treatment to monitor progress and inform decision-making [64]. Improving the standardisation and reporting of symptom severity outcomes in future studies would strengthen the evidence base for pharmacist-led interventions in MH settings.

Medication adherence was improved in all seven studies in which it was studied [33, 36, 37, 42–44, 50]. These findings are in line with high-quality studies that have demonstrated that pharmacist-led interventions, including education, counselling, and collaborative care models, are associated with significant improvements in medication adherence across a range of MH conditions and settings [65–67]. However, a key limitation in comparing adherence outcomes across studies is the variability in measurement. Among these seven studies, five different measurement tools were used, including MPR, the MMAS, the MARS, prescription refill tracking, and patient self-reported medication adherence [68–72]. This heterogeneity, of subjective and objective measures, complicates interpretation and reduces the ability to directly compare effect sizes or draw consistent conclusions across interventions. Standardising adherence measurement methods in future research would help strengthen the evidence base and better characterise the impact of pharmacists on this important clinical outcome.

Adherence to physical health guidelines remains a persistent challenge in psychiatric populations, despite well-documented risks of comorbid physical conditions [73]. Evidence suggests that individuals with SMI often receive suboptimal physical health surveillance [74]. This may be, in part, because psychiatric services prioritise the management of MH symptoms, and clinicians may lack the time, resources, or infrastructure to conduct further physical/laboratory assessments [5, 75]. The five studies that evaluated physical health monitoring reported that pharmacist involvement supported better adherence to recommended monitoring protocols, including laboratory investigations and physical assessments [31, 35, 37, 38, 52]. These findings are consistent with broader research highlighting the pharmacist’s role in improving guideline-based monitoring in chronic disease management, particularly in settings where care fragmentation or resource constraints limit comprehensive service delivery [75, 76]. As physical health outcomes are increasingly prioritised in MH policy and care models, integrating pharmacists into psychiatric teams may represent a feasible and effective strategy to mitigate long-term morbidity in this vulnerable population [74, 77].

Pharmacist‑led interventions resulted in improvements in PROMs in two of four studies [41, 42]. Two studies reported on treatment satisfaction with one study reporting a statistically significant improvement between pharmacist involved care versus usual care and a second study reporting that treatment satisfaction scores were high in both pharmacist-led and psychiatrist-led groups [36, 42]. These results are consistent with previous findings on the impact of pharmacist-led interventions in non-psychiatry specific outpatient settings [78]. Malham et al*.* reported that the four studies included in their review that investigated the impact of pharmacists on patient's satisfaction showed beneficial results [42, 79–81]. These findings suggest that pharmacist involvement may enhance the patient experience of care, supporting the value of collaborative models that prioritise both clinical outcomes and patient-centred measures.

The variety of pharmacist roles identified across the included studies reflects the growing recognition of their clinical and collaborative potential in outpatient MH care. While medication-related counselling and adherence support were the most described activities, many pharmacists also contributed to treatment planning. Importantly, several studies extended the pharmacist’s role to include clinical assessments and lifestyle counselling, indicating a more holistic and patient-centred approach [31, 32, 35, 37, 41, 45–49, 51–53]. In a small number of studies, pharmacists were involved in complex clinical tasks such as monitoring treatment effects [32, 34, 35, 52, 53] and administering LAI antipsychotics [31]. These findings are consistent with a broader shift toward integrating pharmacists into MH MDT, where they are increasingly recognised not only as medication experts but as active participants in direct patient care [15, 54, 76]. As healthcare systems continue to prioritise community-based psychiatric care, the diversity of pharmacist roles observed here underscores the need for clear role definitions, appropriate training, and supportive policy frameworks to fully leverage pharmacists’ clinical expertise.

From an implementation perspective, the heterogeneity observed across (i) study designs, (ii) pharmacist roles, and (iii) outcome measures, limited comparability and precluded meta-analysis. This highlights the need for greater standardisation in future service implementation and evaluation. Variation in medication adherence and symptom assessment tools further reduced comparability across studies. Clear role definition, alignment with existing outpatient mental health care pathways, and use of consistent, validated outcome measures, may support more effective implementation and evaluation of pharmacist-led interventions.

### Limitations

Many included studies did not explicitly report the diagnostic classification system used, which limited the ability to stratify findings by specific diagnostic criteria. Studies focusing on behavioural and psychological symptoms of dementia were excluded, as dementia-related care in the Irish healthcare context is primarily managed through geriatric medicine, primary-care and neurology services rather than specialist outpatient psychiatry. In addition, the definition of outpatient mental health settings used in this review was informed by the Irish healthcare system and focused on clinic-based services delivered through hospital outpatient departments and specialist outpatient mental health clinics. Consequently, settings considered outpatient in some countries, e.g. nursing homes, primary-care or residential care facilities, were not included, which may limit the generalizability of the findings.

## Conclusion

This systematic review provides an evidence-based foundation for the inclusion of pharmacists in outpatient psychiatric services. Their inclusion is consistently associated with improvements across multiple outcome domains, including treatment-related outcomes, medication adherence, and monitoring. The findings reinforce the growing recognition that pharmacists are integral members of psychiatric MDT, contributing not only to safer and more effective medication use but also to enhanced patient satisfaction and quality of care.

Despite positive trends, evidence remains limited by methodological heterogeneity, inconsistent outcome measures, and variation in role definition across settings. Standardisation in the application of clinical, adherence and symptom-related measurement tools would strengthen the comparability and generalisability of future research. Similarly, clearer description of pharmacists’ responsibilities within MDT would facilitate replication and scalability of successful models.

The diversity of pharmacist-led activities; from medication counselling to physical health monitoring and clinical assessments, highlights the profession’s capacity and competency to support holistic, patient-centred MH care. However, to fully realise this potential, future research should focus on developing and testing standardised models for pharmacist integration in outpatient MH services. Implementation-focused studies examining cost-effectiveness, patient experiences, and interprofessional collaboration will be essential to translate this evidence into policy and practice.

## Supplementary Information

Below is the link to the electronic supplementary material.

Supplementary file1 (DOCX 58 KB)

Supplementary file2 (DOCX 35 KB)

Supplementary file3 (DOCX 88 KB)

## Data Availability

Not applicable. This manuscript does not report data generation or analysis.

## References

[1] Mental disorders [Internet]. [cited 2025 Sept 30]. https://www.who.int/news-room/fact-sheets/detail/mental-disorders. Accessed 30 Sept 2025

[2] Over a billion people living with mental health conditions – services require urgent scale-up [Internet]. [cited 2025 Sept 30]. https://www.who.int/news/item/02-09-2025-over-a-billion-people-living-with-mental-health-conditions-services-require-urgent-scale-up. Accessed 30 Sept 2025

[3] Rehm J, Shield KD. Global burden of disease and the impact of mental and addictive disorders. Curr Psychiatry Rep. 2019;21:10. 10.1007/s11920-019-0997-0. 30729322 10.1007/s11920-019-0997-0

[4] Walker ER, McGee RE, Druss BG. Mortality in mental disorders and global disease burden implications: a systematic review and meta-analysis. JAMA Psychiat. 2015;72:334–41. 10.1001/jamapsychiatry.2014.2502. 10.1001/jamapsychiatry.2014.2502 PMC4461039 25671328

[5] DE Hert M, Correll CU, Bobes J, et al. Physical illness in patients with severe mental disorders. I. Prevalence, impact of medications and disparities in health care. World Psychiatry. 2011;10:52–77. 10.1002/j.2051-5545.2011.tb00014.x. 21379357 10.1002/j.2051-5545.2011.tb00014.x PMC3048500

[6] Vancampfort D, Firth J, Schuch FB, et al. Sedentary behavior and physical activity levels in people with schizophrenia, bipolar disorder and major depressive disorder: a global systematic review and meta-analysis. World Psychiatry. 2017;16:308–15. 10.1002/wps.20458. 28941119 10.1002/wps.20458 PMC5608847

[7] El-Den S, Collins JC, Chen TF, et al. Pharmacists’ roles in mental healthcare: Past, present and future. Pharmacy Pract. 2021;19:2545. 10.18549/PharmPract.2021.3.2545. 10.18549/PharmPract.2021.3.2545 PMC8456342 34621456

[8] NICE guidelines developed by NCCMH [Internet]. www.rcpsych.ac.uk. [cited 2026 Mar 10]. https://www.rcpsych.ac.uk/improving-care/nccmh/clinical-guideline-development/nice-guidelines. Accessed 10 Mar 2026

[9] Leichsenring F, Steinert C, Rabung S, et al. The efficacy of psychotherapies and pharmacotherapies for mental disorders in adults: an umbrella review and meta‐analytic evaluation of recent meta‐analyses. World Psychiatry. 2022;21:133–45. 10.1002/wps.20941. 35015359 10.1002/wps.20941 PMC8751557

[10] Mareya S, Watts MC, Zhao L, et al. Exploring the stepped care model in delivering primary mental health services—a scoping review. Int J Ment Health Nurs. 2024. 10.1111/inm.13427. 39301997 10.1111/inm.13427

[11] Thornicroft G, Deb T, Henderson C. Community mental health care worldwide: current status and further developments. World Psychiatry. 2016;15:276–86. 10.1002/wps.20349. 27717265 10.1002/wps.20349 PMC5032514

[12] Slade M, Amering M, Oades L. Recovery: an international perspective. Epidemiol Psichiatr Soc. 2008;17:128–37. 10.1017/s1121189x00002827. 18589629 10.1017/s1121189x00002827

[13] Lefurgey S, Detillieux S, Shaheen A, et al. Person-centered care: learning from the evolution of mental health care. Encyclopedia. 2025;5:29. 10.3390/encyclopedia5010029.

[14] Comprehensive Mental Health Action Plan 2013–2030 [Internet]. [cited 2025 Sept 30]. https://www.who.int/publications/i/item/9789240031029. Accessed 30 Sept 2025

[15] Stuhec M, Hahn M, Taskova I, et al. Clinical pharmacy services in mental health in Europe: a commentary paper of the European Society of Clinical Pharmacy Special Interest Group on Mental Health. Int J Clin Pharm. 2023;45:1286. 10.1007/s11096-023-01643-4. 37755642 10.1007/s11096-023-01643-4 PMC10600282

[16] Advancing the role of pharmacists to meet changing patient and health system needs [Internet]. [cited 2025 Sept 30]. https://www.who.int/europe/news-room/feature-stories/item/advancing-the-role-of-pharmacists-to-meet-changing-patient-and-health-system-needs. Accessed 30 Sept 2025

[17] Boudreau DM, Capoccia KL, Sullivan SD, et al. Collaborative care model to improve outcomes in major depression. Ann Pharmacother. 2002;36:585–91. 10.1345/aph.1A259. 11918503 10.1345/aph.1A259

[18] Buist E, McLelland R, Rushworth GF, et al. An evaluation of mental health clinical pharmacist independent prescribers within general practice in remote and rural Scotland. Int J Clin Pharm. 2019;41:1138–42. 10.1007/s11096-019-00897-1. 31493208 10.1007/s11096-019-00897-1

[19] Alsaeed BA, Hall J, Keers RN. Evaluating pharmacist independent prescribing for patients with mental illness in community care: a qualitative study. Front Psychiatry. 2025. 10.3389/fpsyt.2025.1637132. 40989033 10.3389/fpsyt.2025.1637132 PMC12450940

[20] Stuhec M, Lah L. Clinical pharmacist interventions in elderly patients with mental disorders in primary care focused on psychotropics: a retrospective pre-post observational study. Ther Adv Psychopharmacol. 2021;11:20451253211011010. 10.1177/20451253211011007. 34025980 10.1177/20451253211011007 PMC8072848

[21] Javedh S, Gm RP, Bs S. A systematic review on the impact of clinical pharmacist interventions in patients with mental health disorders. Clin Schizophr Relat Psychoses. 2020;14(3). 10.3371/CSRP.JSGR.112420

[22] Bell S, McLachlan AJ, Chen TF, et al. Community pharmacy services to optimise the use of medications for mental illness: a systematic review. Aust N Z Health Policy. 2005. 10.1071/hp050229. 10.1186/1743-8462-2-29 PMC1345690 16336646

[23] Davis B, Qian J, Ngorsuraches S, et al. The clinical impact of pharmacist services on mental health collaborative teams: a systematic review. J Am Pharm Assoc. 2020;60:S44-53. 10.1016/j.japh.2020.05.006. 10.1016/j.japh.2020.05.006 PMC7529835 32600986

[24] Ng R, El-Den S, Stewart V, et al. Pharmacist-led interventions for people living with severe and persistent mental illness: a systematic review. Aust N Z J Psychiatry. 2022;56:1080–103. 10.1177/00048674211048410. 34560826 10.1177/00048674211048410

[25] Stuhec M, Zelko E. A collaborative care model between general practitioners and clinical pharmacists in a community health centre setting in depression treatment. Psychiatr Danub Croatia. 2021;33:1261–6. 35503938

[26] Page MJ, McKenzie JE, Bossuyt PM, et al. The PRISMA 2020 statement: an updated guideline for reporting systematic reviews. BMJ. 2021;372:n71. 10.1136/bmj.n71. 33782057 10.1136/bmj.n71 PMC8005924

[27] Cochrane Handbook for Systematic Reviews of Interventions (current version) | Cochrane [Internet]. [cited 2026 Mar 3]. https://www.cochrane.org/authors/handbooks-and-manuals/handbook/current. Accessed 3 Mar 2026

[28] PROSPERO [Internet]. [cited 2025 Sept 9]. https://www.crd.york.ac.uk/PROSPERO/view/CRD42025629212. Accessed 9 Sept 2025

[29] RoB 2: A revised Cochrane risk-of-bias tool for randomized trials | Cochrane Bias [Internet]. [cited 2025 Sept 8]. https://methods.cochrane.org/bias/resources/rob-2-revised-cochrane-risk-bias-tool-randomized-trials. Accessed 8 Sept 2025

[30] ROBINS-I tool | Cochrane Methods [Internet]. [cited 2025 Sept 8]. https://methods.cochrane.org/robins-i. Accessed 8 Sept 2025

[31] Chang H“, Vaughn LM, Liu D. Rural ambulatory care pharmacists providing in-clinic and home visit services improve adherence to long-acting injectable antipsychotics. Ment Health Clin. 2024;14:229–32. 10.9740/mhc.2024.06.229. 38835813 10.9740/mhc.2024.06.229 PMC11147658

[32] Gregorian T, Bradley K, Campbell S, et al. Design, implementation, and evaluation of a pharmacist-led outpatient benzodiazepine-tapering clinic. J Am Pharm Assoc. 2023;63:409–15. 10.1016/j.japh.2022.09.025. 10.1016/j.japh.2022.09.025 PMC9945842 36564330

[33] Lindell VA, Stencel NL, Ives RC, et al. A pilot evaluating clinical pharmacy services in an ambulatory psychiatry setting. Psychopharmacol Bull. 2018;48:18–28. 29713097 10.64719/pb.4562 PMC5875359

[34] Lobeck F, Traxler WT, Bobinet DD. The cost-effectiveness of a clinical pharmacy service in an outpatient mental health clinic. Hosp Commun Psychiatry. 1989;40:643–5. 10.1176/ps.40.6.643 2500390

[35] Mailloux LM, Haas MT, Larew JM, et al. Development and implementation of a physician-pharmacist collaborative practice model for provision and management of buprenorphine/naloxone. Ment Health Clin. 2021;11:35–9. 10.9740/mhc.2021.01.035. 33505825 10.9740/mhc.2021.01.035 PMC7800330

[36] Mattle AG, Aladeen T, Blondell RD, et al. Evaluating outcomes of a clinical pharmacist medication management program in a multidisciplinary practice for outpatient buprenorphine treatment of opioid use disorder. JACCP J Am Coll Clin Pharm. 2021;4:424–34. 10.1002/jac5.1405.

[37] Olson C, Vallabh A. A retrospective comparison of pharmacist and psychiatrist-led medication management clinics in an outpatient setting. JACCP J Am Coll Clin Pharm. 2024;7:90–4. 10.1002/jac5.1889.

[38] Pohl L, El-Kurdi R, Selinger R, et al. Implementation of a community-based pharmacist-run attention deficit hyperactivity disorder clinic in a college health center. J Am Pharm Assoc JAPhA. 2021;61:S178–83. 10.1016/j.japh.2021.01.029. 10.1016/j.japh.2021.01.029 33676837

[39] Vickery PB, Godwin K, Roach JK. Increasing access to psychiatric care during the COVID-19 pandemic through mental health clinical pharmacy specialist services. Ment Health Clin. 2023;13:176–82. 10.9740/mhc.2023.08.176. 37860585 10.9740/mhc.2023.08.176 PMC10583258

[40] Schneiderhan ME, Shuster SM, Davey CS. Twelve-month prospective randomized study of pharmacists utilizing point-of-care testing for metabolic syndrome and related conditions in subjects prescribed antipsychotics. Prim Care Companion CNS Disord. 2014. 10.4088/PCC.14m01669. 25667811 10.4088/PCC.14m01669 PMC4321016

[41] Al-Saffar N, Abdulkareem A, Abdulhakeem A, et al. Depressed patients’ preferences for education about medications by pharmacists in Kuwait. Patient Educ Couns. 2008;72:94–101. 10.1016/j.pec.2008.01.027. 18337052 10.1016/j.pec.2008.01.027

[42] Aljumah K, Hassali MA. Impact of pharmacist intervention on adherence and measurable patient outcomes among depressed patients: a randomised controlled study. BMC Psychiatry. 2015;15:219. 10.1186/s12888-015-0605-8. 26376830 10.1186/s12888-015-0605-8 PMC4574071

[43] Mishra A, Krishna GS, Alla S, et al. Impact of pharmacist–psychiatrist collaborative patient education on medication adherence and quality of life (QOL) of bipolar affective disorder (BPAD) patients. Front Pharmacol. 2017. 10.3389/fphar.2017.00722. 29066976 10.3389/fphar.2017.00722 PMC5641349

[44] Mishra A, Sai Krishna G, Sravani A, et al. Impact of pharmacist-led collaborative patient education on medication adherence and quality of life of schizophrenia patients in a tertiary care setting. Bull Fac Pharm Cairo Univ. 2017;55:345–9. 10.1016/j.bfopcu.2017.08.001.

[45] Salazar-Ospina A, Amariles P, Hincapié-García JA, et al. Effectiveness of the dader method for pharmaceutical care on patients with bipolar i disorder: results from the EMDADER-TAB study. J Manag Care Spec Pharm. 2017. 10.18553/jmcp.2017.23.1.74. 28025928 10.18553/jmcp.2017.23.1.74 PMC10398179

[46] Salazar-Ospina A, Amariles P, Hincapié-García JA, et al. Long-term impact of pharmacist intervention in patients with bipolar disorder: extended follow-up to the EMDADER-TAB study. Heliyon. 2020. 10.1016/j.heliyon.2020.e03333. 32072044 10.1016/j.heliyon.2020.e03333 PMC7016228

[47] Sathienluckana T, Unaharassamee W, Suthisisang C, et al. Anticholinergic discontinuation and cognitive functions in patients with schizophrenia: a pharmacist–physician collaboration in the outpatient department. Integr Pharm Res Pract. 2018;7:161–71. 10.2147/IPRP.S176653. 30464898 10.2147/IPRP.S176653 PMC6208936

[48] Singh PA. The impact of clinical pharmacist lead collaborative care on quality of life of the patients with bipolar disorder: a unicenter prospective, randomization study. Indian J Pharm Educ Res. 2017;51:s129–35. 10.5530/ijper.51.2s.59.

[49] Alves L, Carlos J, Rosana M, et al. Assessment of the effectiveness of pharmacotherapy follow-up in patients treated for depression. J Manag Care Pharm. 2013;19:218–27. 10.18553/jmcp.2013.19.3.218. 23537456 10.18553/jmcp.2013.19.3.218 PMC10438347

[50] Mishra A, Kishor MR, Ramesh M. Randomized controlled trial to assess medication adherence and health-related quality of life through a collaborative pharmacist-psychiatrist approach to patient education in patients with depression in India. Front Psychiatry. 2025. 10.3389/fpsyt.2025.1499893. 40071277 10.3389/fpsyt.2025.1499893 PMC11894377

[51] Dorevitch A, Aronzon R, Zilberman L. Medication maintenance of chronic schizophrenic out–patients by a psychiatric clinical pharmacist: 10–year follow–up study. J Clin Pharm Ther. 1993;18:183–6. 10.1111/j.1365-2710.1993.tb00610.x. 8102143 10.1111/j.1365-2710.1993.tb00610.x

[52] Spann G, Austin L, King E. Pharmacists in clozapine clinics improving physical health monitoring. Ment Health Clin. 2022;12:193–8. 10.9740/mhc.2022.06.193. 35801163 10.9740/mhc.2022.06.193 PMC9190272

[53] Fridman GA, Filinger EJ, Presman A. Pharmaceutical care in the follow-up of psychiatric outpatients. Pharm Care Esp. 2006;8:11–7.

[54] Al Shakhori M, Arain S, Abdulsalim S, et al. Effectiveness of a pharmacist-led tele-psychiatric clinic in managing drug-related problems. J Pharm Policy Pract. 2025;18:2460038. 10.1080/20523211.2025.2460038. 39931673 10.1080/20523211.2025.2460038 PMC11809177

[55] Finley PR, Bluml BM, Bunting BA, et al. Clinical and economic outcomes of a pilot project examining pharmacist-focused collaborative care treatment for depression. J Am Pharm Assoc. 2011;51:40–9. 10.1331/JAPhA.2011.09147. 10.1331/JAPhA.2011.09147 21247825

[56] Adler DA, Bungay KM, Wilson IB, et al. The impact of a pharmacist intervention on 6-month outcomes in depressed primary care patients. Gen Hosp Psychiatry. 2004;26:199–209. 10.1016/j.genhosppsych.2003.08.005. 15121348 10.1016/j.genhosppsych.2003.08.005

[57] The future of the mental health workforce [Internet]. Cent. Ment. Health. [cited 2025 Sept 16]. https://www.centreformentalhealth.org.uk/publications/future-mental-health-workforce/. Accessed 16 Sept 2025

[58] Stuhec M, Zorjan K. Clinical pharmacist interventions in ambulatory psychogeriatric patients with excessive polypharmacy. Sci Rep. 2022;12:11387. 10.1038/s41598-022-15657-x. 35794225 10.1038/s41598-022-15657-x PMC9259566

[59] Al-Taani GM, Muflih SM, Al-Azzam SI, et al. Costs saved and avoided from pharmacist interventions to address drug-related problems identified from outpatient clinics in Jordan. PLoS One. 2024;19:e0302287. 10.1371/journal.pone.0302287. 38843244 10.1371/journal.pone.0302287 PMC11156302

[60] Kroenke K, Spitzer RL, Williams JB. The PHQ-9: validity of a brief depression severity measure. J Gen Intern Med. 2001;16:606–13. 10.1046/j.1525-1497.2001.016009606.x. 11556941 10.1046/j.1525-1497.2001.016009606.x PMC1495268

[61] Spitzer RL, Kroenke K, Williams JBW, et al. A brief measure for assessing generalized anxiety disorder: the GAD-7. Arch Intern Med. 2006;166:1092–7. 10.1001/archinte.166.10.1092. 16717171 10.1001/archinte.166.10.1092

[62] Kay SR, Fiszbein A, Opler LA. The positive and negative syndrome scale (PANSS) for schizophrenia. Schizophr Bull. 1987;13:261–76. 10.1093/schbul/13.2.261. 3616518 10.1093/schbul/13.2.261

[63] Newson JJ, Hunter D, Thiagarajan TC. The heterogeneity of mental health assessment. Front Psychiatry. 2020;11:76. 10.3389/fpsyt.2020.00076. 32174852 10.3389/fpsyt.2020.00076 PMC7057249

[64] DSM-5-TR Online Assessment Measures [Internet]. [cited 2025 Sept 17]. https://www.psychiatry.org:443/psychiatrists/practice/dsm/educational-resources/assessment-measures. Accessed 17 Sept 2025

[65] Östbring MJ, Eriksson T, Petersson G, et al. Effects of a pharmaceutical care intervention on clinical outcomes and patient adherence in coronary heart disease: the MIMeRiC randomized controlled trial. BMC Cardiovasc Disord. 2021;21:367. 10.1186/s12872-021-02178-0. 34334142 10.1186/s12872-021-02178-0 PMC8327441

[66] Cahaya N, Kristina SA, Widayanti AW, et al. Pharmacist-led Si-care (schizophrenia care) model to improve medication adherence and symptom management in schizophrenia. Explor Res Clin Soc Pharm. 2024;16:100544. 10.1016/j.rcsop.2024.100544. 39687446 10.1016/j.rcsop.2024.100544 PMC11647222

[67] Wang L, Zhao Y, Han L, et al. Pharmacist-led management model and medication adherence among patients with chronic heart failure: a randomized clinical trial. JAMA Netw Open. 2024;7:e2453976. 10.1001/jamanetworkopen.2024.53976. 39705029 10.1001/jamanetworkopen.2024.53976 PMC11662253

[68] Morisky DE, Ang A, Krousel-Wood M, et al. Retracted: predictive validity of a medication adherence measure in an outpatient setting. J Clin Hypertens. 2008;10:348–54. 10.1111/j.1751-7176.2008.07572.x. 10.1111/j.1751-7176.2008.07572.x PMC2562622 18453793

[69] Kozma CM, Dickson M, Phillips AL, et al. Medication possession ratio: implications of using fixed and variable observation periods in assessing adherence with disease-modifying drugs in patients with multiple sclerosis. Patient Prefer Adherence. 2013;7:509–16. 10.2147/PPA.S40736. 23807840 10.2147/PPA.S40736 PMC3685450

[70] Andrade SE, Kahler KH, Frech F, et al. Methods for evaluation of medication adherence and persistence using automated databases. Pharmacoepidemiol Drug Saf. 2006;15:565–74. 10.1002/pds.1230. 16514590 10.1002/pds.1230

[71] Lam WY, Fresco P. Medication adherence measures: an overview. BioMed Res Int. 2015;2015:217047. 10.1155/2015/217047. 26539470 10.1155/2015/217047 PMC4619779

[72] Thompson K, Kulkarni J, Sergejew AA. Reliability and validity of a new medication adherence rating scale (MARS) for the psychoses. Schizophr Res. 2000;42:241–7. 10.1016/s0920-9964(99)00130-9. 10785582 10.1016/s0920-9964(99)00130-9

[73] Pizzol D, Trott M, Butler L, et al. Relationship between severe mental illness and physical multimorbidity: a meta-analysis and call for action. BMJ Ment Health. 2023;26:e300870. 10.1136/bmjment-2023-300870. 37907331 10.1136/bmjment-2023-300870 PMC10619039

[74] Physical Health of People with Severe Mental illness | Mental Health Commission [Internet]. [cited 2025 Nov 11]. https://www.mhcirl.ie/publications/physical-health-people-severe-mental-illness. Accessed 11 Nov 2025

[75] Bui TNT, Hotham E, Kelly F, et al. Feasibility of a pharmacist-led physical health monitoring for patients on antipsychotic medications: protocol for a longitudinal study. BMJ Open. 2022;12:e059573. 10.1136/bmjopen-2021-059573. 35725265 10.1136/bmjopen-2021-059573 PMC9214376

[76] Goldstone LW, DiPaula BA, Werremeyer A, et al. The role of board-certified psychiatric pharmacists in expanding access to care and improving patient outcomes. Psychiatr Serv. 2021;72:794–801. 10.1176/appi.ps.202000066. 33940946 10.1176/appi.ps.202000066

[77] Sharing the Vision [Internet]. HSE.ie. [cited 2025 Nov 11]. https://www.hse.ie/eng/about/who/mentalhealth/sharing-the-vision/sharing-the-vision.html. Accessed 11 Nov 2025

[78] Bou Malham C, El Khatib S, Cestac P, et al. Impact of pharmacist-led interventions on patient care in ambulatory care settings: a systematic review. Int J Clin Pract. 2021. 10.1111/ijcp.14864. 34523204 10.1111/ijcp.14864

[79] Bernsten C, Björkman I, Caramona M, et al. Improving the well-being of elderly patients via community pharmacy-based provision of pharmaceutical care: a multicentre study in seven European countries. Drugs Aging. 2001;18:63–77. 10.2165/00002512-200118010-00005. 11232739 10.2165/00002512-200118010-00005

[80] Basheti IA, Tadros OKI, Alnajjar MS, et al. Assessing patient satisfaction with the medication management review service delivered in Jordan. J Pharm Health Serv Res. 2019;10:49–55. 10.1111/jphs.12233.

[81] Sorensen L, Stokes JA, Purdie DM, et al. Medication reviews in the community: results of a randomized, controlled effectiveness trial. Br J Clin Pharmacol. 2004;58:648–64. 10.1111/j.1365-2125.2004.02220.x. 15563363 10.1111/j.1365-2125.2004.02220.x PMC1884656

