# Supplementary material for: Evaluating the impact of pharmacists in outpatient mental health settings: a systematic review
Source: Int J Clin Pharm. 2026 Apr 15;48(4):1197–213. doi: 10.1007/s11096-026-02135-x (PMC13369247; doi:10.1007/s11096-026-02135-x)
Supplement: Supplementary file 1 — Supplementary file1 (DOCX 58 KB) [file 11096_2026_2135_MOESM1_ESM.docx]

**Title: Evaluating the Impact of Pharmacists in Outpatient Mental Health Settings: A Systematic Review
Journal: International Journal of Clinical Pharmacy**

**Author Information:**

Muireann Vaughan* (1,2), Maria Donovan (1), Stephen Byrne (1), Fulvio Bedani (3), Sinéad O'Brien (4), Ciaran Halleran (2), Laura J Sahm (1, 2).

1. Pharmaceutical Care Research Group, School of Pharmacy, University College Cork, Cork, Ireland.
2. Pharmacy Department, Mercy University Hospital, Grenville Place, Cork, Ireland.
3. North Lee Mental Health Services, Health Service Executive, Cork, Ireland
4. Cork Mental Health Services, Health Service Executive, Cork, Ireland.

*Corresponding Author e-mail address: [mvaughan@umail.ucc.ie](mailto:mvaughan@umail.ucc.ie)

*Corresponding Author ORCID: 0009-0007-8317-0118

|  | **EMBASE (Elsevier) Search Strategy** |
| --- | --- |
| **Search No.** | **Query** |
| **#28 FINAL SEARCH** | **#4 AND #16 AND #27** |
| #27 | #17 OR #18 OR #19 OR #20 OR #21 OR #22 OR #23 OR #24 OR #25 OR #26 |
| #26 | ('out-of-hospital' NEAR/2 (care OR service* OR clinic*)):ti,ab |
| #25 | (ambulatory NEAR/2 (care OR service* OR clinic*)):ti,ab |
| #24 | outpatient*:ti OR 'day hospital*':ti OR 'mental health clinic*':ti OR 'non-hospital* patient*':ti |
| #23 | outpatient*:ab OR 'day hospital*':ab OR 'mental health clinic*':ab OR 'non-hospital* patient*':ab |
| #22 | 'outpatient department'/exp |
| #21 | 'ambulatory care'/exp |
| #20 | 'outpatient care'/exp |
| #19 | 'outpatient'/exp |
| #18 | 'community mental health service'/exp |
| #17 | 'community mental health center'/exp |
| #16 | #5 OR #6 OR #9 OR #10 OR #11 OR #12 OR #13 OR #14 OR #15 |
| #15 | psychiat*:ti OR psychotropic:ti OR 'alcohol related disorders':ti OR 'psychological disorder*':ti OR 'phobic disorder*':ti OR 'panic disorder*':ti OR 'mental health':ti OR 'mental illness*':ti OR 'adjustment disorder*':ti OR depress*:ti OR 'mood disorder*':ti OR 'neurotic disorder*':ti OR 'affective disorder*':ti OR anxiety:ti OR 'dissociative disorder*':ti OR 'factitio#s disorder*':ti OR 'elimination disorder*':ti OR 'somatoform disorder*':ti OR delirium:ti OR delusion:ti OR psychos?s:ti OR 'paraphilic disorder*':ti OR 'impulse control disorder*':ti OR psychotic:ti OR 'paranoid disorder':ti OR schizo*:ti OR 'substance abuse':ti OR 'substance misuse':ti OR 'substance dependence':ti OR 'substance use disorder*':ti OR 'eating disorder*':ti OR anorexi*:ti OR bulimi*:ti OR ptsd:ti OR 'post traumatic stress disorder':ti OR ocd:ti OR 'obsessive compulsive disorder':ti OR 'personality disorder*':ti OR 'addictive behaviour':ti OR catatonia:ti |
| #14 | psychiat*:ab OR psychotropic:ab OR 'alcohol related disorders':ab OR 'psychological disorder*':ab OR 'phobic disorder*':ab OR 'panic disorder*':ab OR 'mental health':ab OR 'mental illness*':ab OR 'adjustment disorder*':ab OR depress*:ab OR 'mood disorder*':ab OR 'neurotic disorder*':ab OR 'affective disorder*':ab OR anxiety:ab OR 'dissociative disorder*':ab OR 'factitio#s disorder*':ab OR 'elimination disorder*':ab OR 'somatoform disorder*':ab OR delirium:ab OR delusion:ab OR psychos?s:ab OR 'paraphilic disorder*':ab OR 'impulse control disorder*':ab OR psychotic:ab OR 'paranoid disorder':ab OR schizo*:ab OR 'substance abuse':ab OR 'substance misuse':ab OR 'substance dependence':ab OR 'substance use disorder*':ab OR 'eating disorder*':ab OR anorexi*:ab OR bulimi*:ab OR ptsd:ab OR 'post traumatic stress disorder':ab OR ocd:ab OR 'obsessive compulsive disorder':ab OR 'personality disorder*':ab OR 'addictive behaviour':ab OR catatonia:ab |
| #13 | 'psychotropic agent'/exp |
| #12 | 'enuresis'/exp |
| #11 | 'substance abuse'/exp |
| #10 | 'catatonia'/exp |
| #9 | #7 NOT #8 |
| #8 | 'dementia'/exp |
| #7 | 'mental disease'/exp |
| #6 | 'mental health service'/exp |
| #5 | 'psychiatry'/exp |
| #4 | #1 OR #2 OR #3 |
| #3 | pharmacist*:ab OR pharmacy:ab OR pharmacies:ab OR 'pharmaceutical care':ab OR pharmacist*:ti OR pharmacy:ti OR pharmacies:ti OR 'pharmaceutical care':ti |
| #2 | 'clinical pharmacist'/exp |
| #1 | 'pharmacist'/exp |

|  | **CINAHL (EBSCO) Search Strategy** |
| --- | --- |
| **Search No.** | **Query** |
| **S32 FINAL SEARCH** | **S22 AND S29 AND S31** |
| S31 | S1 OR S2 OR S30 |
| S30 | (MH "Pharmacy, Retail") |
| S29 | S23 OR S24 OR S25 OR S26 OR S27 OR S28 |
| S28 | TI ( Outpatient* OR "day hospital*" OR "mental health clinic*" OR "non-hospital* patient*" OR (("out-of-hospital" OR ambulatory) N2 ( care OR service* OR clinic* ))) OR AB ( Outpatient* OR "day hospital*" OR "mental health clinic*" OR "non-hospital* patient*" OR (("out-of-hospital" OR ambulatory) N2 ( care OR service* OR clinic* ))) |
| S27 | (MH "Ambulatory Care Facilities") |
| S26 | (MH "Ambulatory Care") |
| S25 | (MH "Outpatients") |
| S24 | (MH "Outpatient Service") |
| S23 | (MH "Community Mental Health Services") |
| S22 | S20 OR S21 |
| S21 | TI ( psychiat* OR psychotropic OR "Alcohol Related Disorders" OR "Psychological disorder*" OR "phobic disorder*" OR "panic disorder*" OR "Mental Health" OR "Mental illness*" OR "adjustment disorder*" OR depress* OR "mood disorder*" OR "neurotic disorder*" OR "affective disorder*" OR anxiety OR "dissociative disorder*" OR "factitio#s disorder*" OR "Elimination disorder*" OR "somatoform disorder*" OR delirium OR delusion OR psychos?s OR "paraphilic disorder*" OR "personality disorder*" OR "impulse control disorder*" OR psychotic OR "paranoid disorder" OR schizo* OR "substance abuse" OR "substance misuse" OR "substance dependence" OR "substance use disorder*" OR "eating disorder*" OR anorexi* OR bulimi* OR PTSD OR "post traumatic stress disorder" OR OCD OR "obsessive compulsive disorder" OR "personality disorder*" OR "addictive behaviour" OR catatonia ) OR AB ( psychiat* OR psychotropic OR "Alcohol Related Disorders" OR "Psychological disorder*" OR "phobic disorder*" OR "panic disorder*" OR "Mental Health" OR "Mental illness*" OR "adjustment disorder*" OR depress* OR "mood disorder*" OR "neurotic disorder*" OR "affective disorder*" OR anxiety OR "dissociative disorder*" OR "factitio#s disorder*" OR "Elimination disorder*" OR "somatoform disorder*" OR delirium OR delusion OR psychos?s OR "paraphilic disorder*" OR "personality disorder*" OR "impulse control disorder*" OR psychotic OR "paranoid disorder" OR schizo* OR "substance abuse" OR "substance misuse" OR "substance dependence" OR "substance use disorder*" OR "eating disorder*" OR anorexi* OR bulimi* OR PTSD OR "post traumatic stress disorder" OR OCD OR "obsessive compulsive disorder" OR "personality disorder*" OR "addictive behaviour" OR catatonia ) |
| S20 | (S4 OR S5 OR S6 OR S7 OR S8 OR S11 OR S12 OR S13 OR S14 OR S15 OR S16 OR S17 OR S18 OR S19) |
| S19 | (MH "Psychotropic Drugs+") |
| S18 | (MH "Behavior, Addictive+") |
| S17 | (MH "Enuresis") |
| S16 | (MH "Eating Disorders+") |
| S15 | (MH "Substance Dependence+") |
| S14 | (MH "Substance Use Disorders+") |
| S13 | (MH "Substance Abuse+") |
| S12 | (MH "Catatonia") |
| S11 | s9 NOT s10 |
| S10 | (MH "Dementia") |
| S9 | (MH "Mental Disorders+") |
| S8 | (MH "Persons with Mental Disorders") |
| S7 | (MH "Mental Health Services+") |
| S6 | (MH "Psychiatric Service") |
| S5 | (MH "Psychiatric Patients+") |
| S4 | (MH "Psychiatry") |
| S3 | (S1 OR S2) |
| S2 | TI ( pharmacist* OR pharmacy OR pharmacies OR "pharmaceutical care" ) OR AB ( pharmacist* OR pharmacy OR pharmacies OR "pharmaceutical care" ) |
| S1 | (MH "Pharmacists") |

|  | **MEDLINE (EBSCO) Search Strategy** |
| --- | --- |
| **Search No.** | **Query** |
| **S26 FINAL SEARCH** | **S3 AND S23 AND S25** |
| S25 | S4 OR S5 OR S6 OR S7 OR S10 OR S11 OR S12 OR S13 OR S14 OR S15 OR S16 OR S24 |
| S24 | (MH "Psychotropic Drugs+") |
| S23 | S17 OR S18 OR S19 OR S20 OR S21 OR S22 |
| S22 | TI ( Outpatient* OR "day hospital*" OR "mental health clinic*" OR "non-hospital* patient*" OR (("out-of-hospital" OR ambulatory) N2 ( care OR service* OR clinic* ))) OR AB ( Outpatient* OR "day hospital*" OR "mental health clinic*" OR "non-hospital* patient*" OR (("out-of-hospital" OR ambulatory) N2 ( care OR service* OR clinic* ))) |
| S21 | (MH "Ambulatory Care Facilities") |
| S20 | (MH "Ambulatory Care") |
| S19 | (MH "Outpatients") |
| S18 | (MH "Outpatient Clinics, Hospital") |
| S17 | (MH "Community Mental Health Services") |
| S16 | TI ( psychiat* OR psychotropic OR "Alcohol Related Disorders" OR "Psychological disorder*" OR "phobic disorder*" OR "panic disorder*" OR "Mental Health" OR "Mental illness*" OR "adjustment disorder*" OR depress* OR "mood disorder*" OR "neurotic disorder*" OR "affective disorder*" OR anxiety OR "dissociative disorder*" OR "factitio#s disorder*" OR "Elimination disorder*" OR "somatoform disorder*" OR delirium OR delusion OR psychos?s OR "paraphilic disorder*" OR "personality disorder*" OR "impulse control disorder*" OR psychotic OR "paranoid disorder" OR schizo* OR "substance abuse" OR "substance misuse" OR "substance dependence" OR "substance use disorder*" OR "eating disorder*" OR anorexi* OR bulimi* OR PTSD OR "post traumatic stress disorder" OR OCD OR "obsessive compulsive disorder" OR "personality disorder*" OR "addictive behaviour" OR catatonia ) OR AB ( psychiat* OR psychotropic OR "Alcohol Related Disorders" OR "Psychological disorder*" OR "phobic disorder*" OR "panic disorder*" OR "Mental Health" OR "Mental illness*" OR "adjustment disorder*" OR depress* OR "mood disorder*" OR "neurotic disorder*" OR "affective disorder*" OR anxiety OR "dissociative disorder*" OR "factitio#s disorder*" OR "Elimination disorder*" OR "somatoform disorder*" OR delirium OR delusion OR psychos?s OR "paraphilic disorder*" OR "personality disorder*" OR "impulse control disorder*" OR psychotic OR "paranoid disorder" OR schizo* OR "substance abuse" OR "substance misuse" OR "substance dependence" OR "substance use disorder*" OR "eating disorder*" OR anorexi* OR bulimi* OR PTSD OR "post traumatic stress disorder" OR OCD OR "obsessive compulsive disorder" OR "personality disorder*" OR "addictive behaviour" OR catatonia ) |
| S15 | (MH "Behavior, Addictive+") |
| S14 | (MH "Enuresis") |
| S13 | (MH "Feeding and Eating Disorders+") |
| S12 | (MH "Substance-Related Disorders+") |
| S11 | (MH "Catatonia") |
| S10 | s8 NOT s9 |
| S9 | (MH "Dementia") |
| S8 | (MH "Mental Disorders+") |
| S7 | (MH "Mentally Ill Persons") |
| S6 | (MH "Mental Health Services+") |
| S5 | (MH "Community Psychiatry") |
| S4 | (MH "Psychiatry") |
| S3 | (S1 OR S2) |
| S2 | TI ( pharmacist* OR pharmacy OR pharmacies OR "pharmaceutical care" ) OR AB (pharmacist* OR pharmacy OR pharmacies OR "pharmaceutical care" ) |
| S1 | (MH "Pharmacists") |

|  | **PsycINFO (EBSCO) Search Strategy** |
| --- | --- |
| **Seach No.** | **Query** |
| **S24 FINAL SEARCH** | **S3 AND S21 AND S23** |
| S23 | S4 OR S5 OR S6 OR S7 OR S8 OR S11 OR S12 OR S13 OR S14 OR S22 |
| S22 | DE "Psychotropic Drugs" OR DE "Antidepressant Drugs" OR DE "Anxiolytic Drugs" OR DE "Psychedelic Drugs" OR DE "Psychotomimetic Drugs" |
| S21 | S15 OR S16 OR S17 OR S18 OR S19 OR S20 |
| S20 | (DE "outpatient treatment") |
| S19 | TI ( Outpatient* OR "day hospital*" OR "mental health clinic*" OR "non-hospital* patient*" OR (("out-of-hospital" OR ambulatory) N2 ( care OR service* OR clinic* ))) OR AB ( Outpatient* OR "day hospital*" OR "mental health clinic*" OR "non-hospital* patient*" OR (("out-of-hospital" OR ambulatory) N2 ( care OR service* OR clinic* ))) |
| S18 | (DE "Psychiatric Clinics") |
| S17 | (DE "Outpatients") |
| S16 | (DE "Community Mental Health Centers") |
| S15 | (DE "Community Mental Health Services") |
| S14 | TI ( psychiat* OR psychotropic OR "Alcohol Related Disorders" OR "Psychological disorder*" OR "phobic disorder*" OR "panic disorder*" OR "Mental Health" OR "Mental illness*" OR "adjustment disorder*" OR depress* OR "mood disorder*" OR "neurotic disorder*" OR "affective disorder*" OR anxiety OR "dissociative disorder*" OR "factitio#s disorder*" OR "Elimination disorder*" OR "somatoform disorder*" OR delirium OR delusion OR psychos?s OR "paraphilic disorder*" OR "personality disorder*" OR "impulse control disorder*" OR psychotic OR "paranoid disorder" OR schizo* OR "substance abuse" OR "substance misuse" OR "substance dependence" OR "substance use disorder*" OR "eating disorder*" OR anorexi* OR bulimi* OR PTSD OR "post traumatic stress disorder" OR OCD OR "obsessive compulsive disorder" OR "personality disorder*" OR "addictive behaviour" OR catatonia ) OR AB ( psychiat* OR psychotropic OR "Alcohol Related Disorders" OR "Psychological disorder*" OR "phobic disorder*" OR "panic disorder*" OR "Mental Health" OR "Mental illness*" OR "adjustment disorder*" OR depress* OR "mood disorder*" OR "neurotic disorder*" OR "affective disorder*" OR anxiety OR "dissociative disorder*" OR "factitio#s disorder*" OR "Elimination disorder*" OR "somatoform disorder*" OR delirium OR delusion OR psychos?s OR "paraphilic disorder*" OR "personality disorder*" OR "impulse control disorder*" OR psychotic OR "paranoid disorder" OR schizo* OR "substance abuse" OR "substance misuse" OR "substance dependence" OR "substance use disorder*" OR "eating disorder*" OR anorexi* OR bulimi* OR PTSD OR "post traumatic stress disorder" OR OCD OR "obsessive compulsive disorder" OR "personality disorder*" OR "addictive behaviour" OR catatonia ) |
| S13 | (DE "Urinary Incontinence") |
| S12 | (DE "Catatonia") |
| S11 | s9 NOT s10 |
| S10 | (DE "Dementia" OR DE "AIDS Dementia Complex" OR DE "Alzheimer's Disease" OR DE "Dementia with Lewy Bodies" OR DE "Frontotemporal Lobar Degeneration" OR DE "Presenile Dementia" OR DE "Pseudodementia" OR DE "Senile Dementia" OR DE "Vascular Dementia"") |
| S9 | (DE "Mental Disorders" OR DE "Affective Disorders" OR DE "Anxiety Disorders" OR DE "Behavior Disorders" OR DE "Bipolar Disorder" OR DE "Borderline States" OR DE "Chronic Mental Illness" OR DE "Dissociative Disorders" OR DE "Eating Disorders" OR DE "Gender Dysphoria" OR DE "Mental Disorders due to General Medical Conditions" OR DE "Neurocognitive Disorders" OR DE "Neurodevelopmental Disorders" OR DE "Neurosis" OR DE "Obsessive Compulsive Disorder" OR DE "Paraphilias" OR DE "Personality Disorders" OR DE "Psychosis" OR DE "Serious Mental Illness" OR DE "Sleep Wake Disorders" OR DE "Somatoform Disorders" OR DE "Stress and Trauma Related Disorders" OR DE "Substance Related and Addictive Disorders" OR DE "Thought Disorders" OR DE "Affective Disorders" OR DE "Disruptive Mood Dysregulation Disorder" OR DE "Major Depression" OR DE "Persistent Depressive Disorder" OR DE "Premenstrual Dysphoric Disorder" OR DE "Anxiety Disorders" OR DE "Castration Anxiety" OR DE "Generalized Anxiety Disorder" OR DE "Panic Attack" OR DE "Panic Disorder" OR DE "Phobias" OR DE "Selective Mutism" OR DE "Separation Anxiety Disorder" OR DE "Social Anxiety Disorder" OR DE "Behavior Disorders" OR DE "Conduct Disorder" OR DE "Disruptive Behavior Disorders" OR DE "Emotional and Behavioral Disorders" OR DE "Impulse Control Disorders" OR DE "Kleptomania" OR DE "Oppositional Defiant Disorder" OR DE "Pyromania" OR DE "Self-Destructive Behavior" OR DE "Bipolar Disorder" OR DE "Bipolar I Disorder" OR DE "Bipolar II Disorder" OR DE "Cyclothymic Disorder" OR DE "Mania" OR DE "Chronic Mental Illness" OR DE "Chronic Psychosis" OR DE "Dissociative Disorders" OR DE "Depersonalization" OR DE "Depersonalization/Derealization Disorder" OR DE "Dissociative Amnesia" OR DE "Dissociative Fugue" OR DE "Dissociative Identity Disorder" OR DE "Eating Disorders" OR DE "Anorexia Nervosa" OR DE "Avoidant/Restrictive Food Intake Disorder" OR DE "Binge Eating Disorder" OR DE "Bulimia" OR DE "Feeding Disorders" OR DE "Hyperphagia" OR DE "Kleine Levin Syndrome" OR DE "Orthorexia" OR DE "Pica" OR DE "Purging (Eating Disorders)" OR DE "Rumination (Eating)" OR DE "Neurocognitive Disorders" OR DE "Auditory Processing Disorder" OR DE "Delirium" OR DE "Dementia" OR DE "Memory Disorders" OR DE "Mild Cognitive Impairment" OR DE "Neurodevelopmental Disorders" OR DE "Attention Deficit Disorder" OR DE "Autism Spectrum Disorders" OR DE "Dyspraxia" OR DE "Intellectual Development Disorder" OR DE "Learning Disorders" OR DE "Social Communication Disorder" OR DE "Speech Sound Disorder" OR DE "Stereotypic Movement Disorder" OR DE "Stuttering" OR DE "Tic Disorders" OR DE "Neurosis" OR DE "Childhood Neurosis" OR DE "Experimental Neurosis" OR DE "Traumatic Neurosis" OR DE "Obsessive Compulsive Disorder" OR DE "Body Dysmorphic Disorder" OR DE "Excoriation Disorder" OR DE "Hoarding Disorder" OR DE "Koro" OR DE "Trichotillomania" OR DE "Paraphilias" OR DE "Exhibitionism" OR DE "Fetishism" OR DE "Pedophilia" OR DE "Sexual Masochism" OR DE "Sexual Sadism" OR DE "Voyeurism" OR DE "Personality Disorders" OR DE "Antisocial Personality Disorder" OR DE "Avoidant Personality Disorder" OR DE "Borderline Personality Disorder" OR DE "Dependent Personality Disorder" OR DE "Histrionic Personality Disorder" OR DE "Narcissistic Personality Disorder" OR DE "Obsessive Compulsive Personality Disorder" OR DE "Paranoid Personality Disorder" OR DE "Passive Aggressive Personality Disorder" OR DE "Sadomasochistic Personality" OR DE "Schizoid Personality Disorder" OR DE "Schizotypal Personality Disorder" OR DE "Psychosis" OR DE "Affective Psychosis" OR DE "Alcohol Induced Psychotic Disorders" OR DE "Brief Psychotic Disorder" OR DE "Capgras Syndrome" OR DE "Childhood Onset Psychosis" OR DE "Chronic Psychosis" OR DE "Delusional Disorder" OR DE "Experimental Psychosis" OR DE "Hallucinosis" OR DE "Paranoid Psychosis" OR DE "Postpartum Psychosis" OR DE "Reactive Psychosis" OR DE "Schizophrenia" OR DE "Substance Induced Psychotic Disorders" OR DE "Sleep Wake Disorders" OR DE "Hypersomnia" OR DE "Insomnia" OR DE "Narcolepsy" OR DE "Parasomnias" OR DE "Sleep Apnea" OR DE "Somatoform Disorders" OR DE "Body Dysmorphic Disorder" OR DE "Conversion Disorder" OR DE "Factitious Disorders" OR DE "Illness Anxiety Disorder" OR DE "Neurasthenia" OR DE "Somatization Disorder" OR DE "Somatoform Pain Disorder" OR DE "Stress and Trauma Related Disorders" OR DE "Acute Stress Disorder" OR DE "Adjustment Disorders" OR DE "Attachment Disorders" OR DE "Disinhibited Social Engagement Disorder" OR DE "Posttraumatic Stress Disorder" OR DE "Prolonged Grief Disorder" OR DE "Substance Related and Addictive Disorders" OR DE "Addiction" OR DE "Nonsubstance Related Addictions" OR DE "Substance Use Disorder" OR DE "Thought Disorders" OR DE "Confabulation" OR DE "Delusions" OR DE "Fantasies (Thought Disturbances)" OR DE "Fragmentation (Schizophrenia)" OR DE "Judgment Disturbances" OR DE "Magical Thinking" OR DE "Obsessions" OR DE "Perseveration"))))) |
| S8 | (DE "Psychiatric Patients") |
| S7 | (DE "Mental Health Services" OR DE "College Mental Health Services" OR DE "Community Mental Health Services" OR DE "Mental Health Programs") |
| S6 | (DE "Psychiatric Symptoms" OR DE "Acting Out" OR DE "Anhedonia" OR DE "Anxiety" OR DE "Body Rocking" OR DE "Catatonia" OR DE "Delirium" OR DE "Delusions" OR DE "Depersonalization" OR DE "Depression (Emotion)" OR DE "Distractibility" OR DE "Emptiness" OR DE "Externalizing Symptoms" OR DE "Hallucinations" OR DE "Hyperactivity" OR DE "Hypomania" OR DE "Internalizing Symptoms" OR DE "Mania" OR DE "Positive and Negative Symptoms" OR DE "Somatization") |
| S5 | (DE "Psychiatric Patients") |
| S4 | DE "Psychiatry" OR DE "Addiction Psychiatry" OR DE "Biological Psychiatry" OR DE "Community Psychiatry" OR DE "Consultation Liaison Psychiatry" OR DE "Forensic Psychiatry" OR DE "Geriatric Psychiatry" OR DE "Military Psychiatry" OR DE "Neuropsychiatry" OR DE "Orthopsychiatry" OR DE "Social Psychiatry" OR DE "Telepsychiatry" OR DE "Transcultural Psychiatry" |
| S3 | (S1 OR S2) |
| S2 | TI ( pharmacist* OR pharmacy OR pharmacies OR "pharmaceutical care" ) OR AB ( pharmacist* OR pharmacy OR pharmacies OR "pharmaceutical care" ) |
| S1 | (DE "Pharmacists") |

|  | **CENTRAL Search Strategy** |
| --- | --- |
| **Search No.** | **Search** |
| **#26 FINAL SEARCH** | **#3 AND #16 AND #23** |
| #25 | #17 OR #18 OR #19 OR #20 OR #21 OR #22 OR #23 OR #24 |
| #24 | (ambulatory NEAR/2 (care OR service* OR clinic*)):ti,ab,kw |
| #23 | ("out-of-hospital" NEAR/2 (care OR service* OR clinic*)):ti,ab,kw |
| #22 | (Outpatient* OR "day hospital" OR "day hospitals" OR "mental health clinic" OR "mental health clinics" OR "non-hospital patient" OR "non-hospitalised patient" OR "non-hospitalized patient" OR "non-hospital patients" OR "non-hospitalised patients" OR "non-hospitalized patients"):ti,ab,kw |
| #21 | MeSH descriptor: [Outpatient Clinics, Hospital] explode all trees |
| #20 | MeSH descriptor: [Ambulatory Care Facilities] explode all trees |
| #19 | MeSH descriptor: [Outpatients] this term only |
| #18 | MeSH descriptor: [Ambulatory Care] this term only |
| #17 | MeSH descriptor: [Community Mental Health Services] this term only |
| #16 | #13 OR #14 OR #15 |
| #15 | (psychiat* OR psychotropic OR "mental health" OR "mental illness" OR depress* OR anxiety OR delirium OR delusion* OR psychosis OR psychoses OR psychotic OR schizo* OR "substance abuse" OR "substance dependence" OR anorexi* OR bulimi* OR PTSD OR OCD OR catatonia):ti,ab,kw |
| #14 | (("alcohol related" OR psychological OR phobic OR panic OR adjustment OR mood OR neurotic OR affective OR dissociative OR factitious OR elimination OR somatoform OR paraphilic OR paranoid OR "substance use" OR eating OR "post traumatic stress" OR "obsessive compulsive" OR personality) NEXT disorder*):ti,ab,kw |
| #13 | #4 OR #5 OR #6 OR #9 OR #10 OR #11 OR #12 |
| #12 | MeSH descriptor: [Behavior, Addictive] explode all trees |
| #11 | MeSH descriptor: [Substance-Related Disorders] explode all trees |
| #10 | MeSH descriptor: [Catatonia] this term only |
| #9 | #7 NOT #8 |
| #8 | MeSH descriptor: [Dementia] explode all trees |
| #7 | MeSH descriptor: [Mental Disorders] explode all trees |
| #6 | MeSH descriptor: [Mentally Ill Persons] explode all trees |
| #5 | MeSH descriptor: [Mental Health Services] explode all trees |
| #4 | MeSH descriptor: [Psychiatry] explode all trees |
| #3 | #1 OR #2 |
| #2 | (pharmacist* OR pharmacy OR pharmacies OR "pharmaceutical care"):ti,ab,kw |
| #1 | MeSH descriptor: [Pharmacists] explode all trees |
