# Supplementary material for: Evaluating the impact of pharmacists in outpatient mental health settings: a systematic review
Source: Int J Clin Pharm. 2026 Apr 15;48(4):1197–213. doi: 10.1007/s11096-026-02135-x (PMC13369247; doi:10.1007/s11096-026-02135-x)
Supplement: Supplementary file 2 — Supplementary file2 (DOCX 35 KB) [file 11096_2026_2135_MOESM2_ESM.docx]

**Title:**Evaluating the Impact of Pharmacists in Outpatient Mental Health Settings: A Systematic Review 
**Journal:** International Journal of Clinical Pharmacy

**Author Information:**

Muireann Vaughan* (1,2), Maria Donovan (1), Stephen Byrne (1), Fulvio Bedani (3), Sinéad O'Brien (4), Ciaran Halleran (2), Laura J Sahm (1, 2). 
 
1. Pharmaceutical Care Research Group, School of Pharmacy, University College Cork, Cork, Ireland. 
2. Pharmacy Department, Mercy University Hospital, Grenville Place, Cork, Ireland. 
3. North Lee Mental Health Services, Health Service Executive, Cork, Ireland 
4. Cork Mental Health Services, Health Service Executive, Cork, Ireland.

*Corresponding Author e-mail address: [mvaughan@umail.ucc.ie](mailto:mvaughan@umail.ucc.ie)

*Corresponding Author ORCID: 0009-0007-8317-0118

Table 1: Summary of reported outcome measures categorised into seven outcome domains

| Patient-reported Outcomes | Symptom-related outcomes | Treatment-related outcomes | Medication Adherence | Cost | Monitoring | Hospitalisations |
| --- | --- | --- | --- | --- | --- | --- |
| Patient Satisfaction with Treatment: TSQM 1.4 | PANNS score | No. of medication related side effects per patient | Medication Adherence: Morisky Medication Adherence Scale | Drug cost per prescription | Urine Drug Screening samples collected | No. of days of hospitalisation for psychiatric reasons |
| Patient Satisfaction with Overall Service | Quality of Life (WHO-BREF SCALE) | Total drug related problems reported | Medication Adherence: Medication Possession Ratio | Personnel cost per prescription | Pertinent Laboratory Monitoring Completed | ED visits + Hospital Admissions per patient |
| Patient using medicine correctly | Brief Psychiatric Rating Scale Score | Patient completely tapered off of benzodiazepines | Medication Adherence: Self-Reported | Total Cost per Prescription (Drug + Personnel) | Heart rate monitoring Completed | No. of Hospitalisations |
| Patient recall how to manage missed doses | Executive function measured by (WCST) perseverative errors | Benzodiazepine dose reduced (tapered down) | Medication Adherence: Medication for Opioid Use Disorder Adherence Issues Addressed |  | Fasting Blood Glucose Monitoring Completed | No. of Emergency Service Consultations |
| Patient engagement with counselling | Severity of Depression: Montgomery–Åsberg Depression Rating Scale | Program Failure | Medication Adherence: Buprenorphine nonadherence identified |  | Fasting Lipid Monitoring Completed |  |
| Patient recall of medicine name | PHQ-9 Scores |  | Medication Adherence: Medication Adherence rating scale |  | Blood Pressure Monitoring Completed |  |
|  | Change in PHQ-9 Score |  | Medication Adherence: Last LAI administration within 2 weeks of the recommended schedule |  |  |  |
|  | Change in GAD-7 Score |  |  |  |  |  |
|  | Beck Depression Inventory score |  |  |  |  |  |
|  | Beck Anxiety Inventory score |  |  |  |  |  |
