# Supplementary material for: Evaluating the impact of pharmacists in outpatient mental health settings: a systematic review
Source: Int J Clin Pharm. 2026 Apr 15;48(4):1197–213. doi: 10.1007/s11096-026-02135-x (PMC13369247; doi:10.1007/s11096-026-02135-x)
Supplement: Supplementary file 3 — Supplementary file3 (DOCX 88 KB) [file 11096_2026_2135_MOESM3_ESM.docx]

**Title: Evaluating the Impact of Pharmacists in Outpatient Mental Health Settings: A Systematic Review
Journal: International Journal of Clinical Pharmacy**

**Author Information:**

Muireann Vaughan* (1,2), Maria Donovan (1), Stephen Byrne (1), Fulvio Bedani (3), Sinéad O'Brien (4), Ciaran Halleran (2), Laura J Sahm (1, 2).

1. Pharmaceutical Care Research Group, School of Pharmacy, University College Cork, Cork, Ireland.
2. Pharmacy Department, Mercy University Hospital, Grenville Place, Cork, Ireland.
3. North Lee Mental Health Services, Health Service Executive, Cork, Ireland
4. Cork Mental Health Services, Health Service Executive, Cork, Ireland.

*Corresponding Author e-mail address: [mvaughan@umail.ucc.ie](mailto:mvaughan@umail.ucc.ie)

*Corresponding Author ORCID: 0009-0007-8317-0118

| **Year,**  **Author,**  **Country** | **Population** | **Duration** | **Inclusion Criteria** | **Exclusion Criteria** | **Participant Diagnoses, mean age (SD) years, % male overall** |
| --- | --- | --- | --- | --- | --- |
| Mishra, 2025, India | Total Sample Size=75  Intervention Group (IG) = 36    Control Group (CG)=39 | 6 months | Patients who visited the psychiatry outpatient department and were of either gender, aged ≥18 years, treated for depression and were literate. | 1. Illiterate patients  2. Patients with pre-existing comorbidities, including other  psychiatric conditions such as cognitive dysfunction or significant illness | Depression, 38.57 ± 12.37, 34.7% |
| **Aim and Pharmacist's Role:** | | | | | |
| To initiate and assess the impact of the pharmacist-psychiatrist collaborative patient education model on medication adherence and the health-related quality of life (HRQoL) in patients with depression.  Patient education was provided to the test group by the clinical pharmacist and was customised for each case based on the severity of the disease. The pharmacist called the patients at least one week and then a day before their next scheduled hospital visit date. | | | | | |
|  |  |  |  |  |  |
|  |  |  |  |  |  |
| **Key Findings:** | | | | | |
| **Medication Adherence:** The collaborative care group showed a statistically significant improvement in medication adherence as measured by the Medication Adherence Rating Scale , with a mean increase of 1.67 ± 0.25 (p< 0.001), compared with a mean increase of 0.69 ± 0.05 (p < 0.05) for the usual care group.  **Symptom Related:** HRQoL scores, as measured by the World Health Organisation Quality of Life-Brief (WHOQOL-BREF) Scale, improved significantly more in the collaborative care group, with a mean increase of 28.01 ± 2.05 (p < 0.001), compared with a mean increase of 12.46 ± 0.26 (p < 0.05) for the usual care group | | | | | |

| **Year,**  **Author,**  **Country** | **Population** | **Duration** | **Inclusion Criteria** | **Exclusion Criteria** | **Participant Diagnoses, mean age (SD) years, % male overall** |
| --- | --- | --- | --- | --- | --- |
| 2024,  Chang,  USA | Total Sample Size = 10  IG (90 days post study start) = 10  CG (90 days prior to study start) = 10  Withdrawals = 0 | 180 days | Patients with active orders for Long Acting Injectable (LAI) antipsychotics within the service. | n/r | n/r IG: n/r . CG: n/r   n/r |
| **Aim and Pharmacist's Role:** | | | | | |
| To examine if pharmacists can improve access and bridge care gaps for patients on LAIs by providing community-based services in addition to traditional clinic-based care.  Non adherent patients were offered follow-up appointments with the pharmacist within the clinics. When patients could not be reached by telephone or were unable to secure transportation for clinic appointments to receive LAI injections, outreach pharmacists administered LAI medications in the home visit setting. During home visits, pharmacists completed a psychiatric assessment, including evaluation of positive and negative symptoms of the patient’s psychiatric disorder, social functioning, potential for suicidality, and presence of behavioural challenges or substance use. Pharmacists also addressed other chronic conditions, new complaints, and health maintenance reminders if applicable. | | | | | |
|  |  |  |  |  |  |
|  |  |  |  |  |  |
| **Key Findings:** | | | | | |
| **Treatment related**: The percentage of days covered by LAI fills increased from an average of 26% to 67% of days covered (p = 0.06).  **Hospitalisations**: Total ED visits related to mental health episodes decreased from 11 to 2 visits (p = 0.03). **Monitoring**: The percentage of patients that had laboratory monitoring completed in the previous year increased from 30% to 80% | | | | | |

| **Year,**  **Author,**  **Country** | **Population** | **Duration** | **Inclusion Criteria** | **Exclusion Criteria** | **Participant Diagnoses, mean age (SD) years, % male overall** |
| --- | --- | --- | --- | --- | --- |
| 2024,  Olson,  USA | Total Sample Size = 221  IG (Pharmacist-led clinic) = 111  CG (Psychiatrist-led clinic) = 110  Withdrawals = 0 | 18 months | 18 years or older,  a documented mental health diagnosis,  engaged in outpatient mental health care with either a psychiatric pharmacist or psychiatrist for medication management | Patients who attended < 3 appointments with their medication management provider during the study period, or  were prescribed controlled substances for their mental health diagnosis,  or were flagged in the electronic medical record as high risk for suicide by the facility's suicide prevention committee. | non- specific,  IG: 55.9 (14.5)  CG: 53.9 (13.8), 87 |
| **Aim and Pharmacist's Role:** | | | | | |
| To assess if a significant difference exists between pharmacist and psychiatrist medication management clinics regarding escalation of care, evidence-based medication prescribing, and medication adherence.  The pharmacist acted as an independent prescriber which granted prescriptive authority for psychotropics, apart from controlled substances. The pharmacist delivered psychiatric care to patients presenting as walk-ins to the clinic, those transferred from a recently retired psychiatrist, and those newly referred for mental health care. | | | | | |
|  |  |  |  |  |  |
|  |  |  |  |  |  |
| **Key Findings:** | | | | | |
| **Medication Adherence**: The pharmacist-led clinic had a significantly higher Medication Possession Ratio (MPR) (p < 0.00001) **Hospitalisations**: Emergency Department (ED) visits and admissions per patient were similar between the pharmacist and psychiatrist groups (0.045 ± 0.248, 0.081 ± 0.307, respectively; p = 0.646) **Monitoring**: The pharmacist-led clinic had a significantly higher rate of completed laboratory monitoring (p = 0.0015). | | | | | |

| **Year,**  **Author,**  **Country** | **Population** | **Duration** | **Inclusion Criteria** | **Exclusion Criteria** | **Participant Diagnoses, mean age (SD) years, % male overall** |
| --- | --- | --- | --- | --- | --- |
| 2023,  Gregorian,  USA | Total Sample Size = 159  Intervention (Enrolled in Pharmacist-led benzodiazepine tapering clinic) = 62  Control (Not Enrolled) = 97  Withdrawals = 0 | 26 months | Adults prescribed long-term benzodiazepines (defined as > 2–4 weeks of use). | Age <18 years Active substance use disorder Active pregnancy History of seizures History of moderate-to-severe benzodiazepine withdrawal, including: Hypertension, moderate/severe anxiety or insomnia, delirium. | n/r  IG: 61 (17.5)  CG: 61 (18.1), 33 |
| **Aim and Pharmacist's Role:** | | | | | |
| To describe the design, implementation, and evaluation of an outpatient pharmacist-led benzodiazepine tapering clinic.  Pharmacists conducted an initial 60-minute intake visit in which they: Reviewed indication of benzodiazepine, medical/medication history, prior use of benzodiazepines, Educated on risks and tapering process, Developed patient-driven tapering plan, Ordered urine drug screens and reviewed Prescription drug monitoring program database, Provided prescription and education for naloxone if concurrent opioid use. Asked patients to sign a Controlled Substances Agreement. Follow-up 30-minute visits (via phone or video): Reviewed adherence, withdrawal symptoms, and made tapering adjustments, Used the Benzodiazepine Withdrawal Symptom Questionnaire, Prescribed adjunct medicines for anxiety/insomnia, Reinforced harm reduction, discussed substance use, and ensured safe refills. Pharmacists were also responsible for: Managing prescriptions, Preventing inappropriate community pharmacy refills and Communicating with primary care and behavioural health teams. | | | | | |
|  |  |  |  |  |  |
|  |  |  |  |  |  |
| **Key Findings:** | | | | | |
| **Treatment Related**: 90% of patients had at least some benzodiazepine tapering when enrolled in the clinic, compared to 41% among not enrolled in the clinic (p < 0.001). 27% of patients enrolled in the clinic were completely tapered off of benzodiazepine in comparison to 4% of those not enrolled (p < 0.001). | | | | | |

| **Year,**  **Author,**  **Country** | **Population** | **Duration** | **Inclusion Criteria** | **Exclusion Criteria** | **Participant Diagnoses, mean age (SD) years, % male overall** |
| --- | --- | --- | --- | --- | --- |
| 2023,  Vickery,  USA | Total = 45  IG (Pharmacist-based care) = 11  CG (Psychiatrist-based care) = 34  Withdrawals = 0 | 10 months | Individuals 18 years of age.  Those who used psychiatric services offered by the clinic.  Those willing to complete a satisfaction survey. | Those who utilized the clinic’s psychiatric services and were unable to complete a satisfaction survey with or without assistance or who were unwilling to complete the survey. | n/r, IG: 40.5 (11.54). CG: 34.6 (6.6) years, 56 |
| **Aim and Pharmacist's Role:** | | | | | |
| To evaluate the addition of a Clinical pharmacist practitioner as an Mental Health Clinical Pharmacy Specialist (MHCPS) to the clinic and the impact this may have on psychiatric care offered.  Pharmacists offered once monthly in-person clinic visits. | | | | | |
| **Key Findings:** | | | | | |
| **Patient Reported**: Treatment Satisfaction scores were high in both groups. The most frequent score among all surveys was 4.8 (p > 0.05) on a 5-point scale, indicating no statistically significant differences between clinician types. | | | | | |

| **Year,**  **Author,**  **Country** | **Population** | **Duration** | **Inclusion Criteria** | **Exclusion Criteria** | **Participant Diagnoses, mean age (SD) years, % male overall** |
| --- | --- | --- | --- | --- | --- |
| 2022,  Spann,  Australia | Total = 61  IG (Pharmacist Clinic) = 33  CG (Usual Care) = 28  Withdrawals = 0 | 1 year | Adult patients (>18 years old) on maintenance clozapine therapy | Changed clinics (owing to relocation) during the study on initiation therapy (first 18 weeks of starting clozapine) or reinitiating clozapine owing to different monitoring requirements and their monitoring also commencing in hospital | Schizophrenia IG: 42.2 (10.2).  CG: 45.7 (9.8), 69 |
| **Aim and Pharmacist's Role:** | | | | | |
| To investigate whether having a pharmacist in a community clozapine clinic would improve adherence to physical health monitoring and whether this would have a positive effect on these physical health outcomes  The pharmacist performed medication reviews every 6 months. These included reviewing physical health monitoring, making recommendations to the provider based on these results and providing lifestyle advice. | | | | | |
|  |  |  |  |  |  |
|  |  |  |  |  |  |
| **Key Findings:** | | | | | |
| **Monitoring**: The pharmacist clinic had statistically higher rates of metabolic monitoring (glucose 48% vs 11%, p < 0.001; lipids 61% vs 7.1%, p < 0.001) and Electrocardiogram (ECG) monitoring (15% vs 0%, p < 0.001). | | | | | |

| **Year,**  **Author,**  **Country** | **Population** | **Duration** | **Inclusion Criteria** | **Exclusion Criteria** | **Participant Diagnoses, mean age (SD) years, % male overall** |
| --- | --- | --- | --- | --- | --- |
| 2021,  Mailloux,  USA | Total = 50  IG(Physician-pharmacist collaborative practice model) = 25  CG (Psychiatrist-only appointments) = 25  Withdrawals = 0 | 5 months | Patients with OUD prescribed buprenorphine/naloxone. Received care at the substance use disorder clinic during the 5-month pilot period | n/r | OUD, IG: 49 (n/r). CG: 47 (n/r), 96 |
| **Description of Pharmacist's Role:** | | | | | |
| To develop and implement a physician-pharmacist collaborative practice model (PPCPM) for managing patients with opioid use disorder (OUD) on buprenorphine/naloxone, aiming to: Reduce psychiatrist workload, Expand access to treatment and Enhance patient care.  The pharmacist conducted patient appointments, assessed and managed patients prescribed buprenorphine/naloxone, identified medication adherence concerns and addressed patient-specific needs. They referred to services and recommended changes to medications. They provided naloxone and performing medication reconciliation. | | | | | |
|  |  |  |  |  |  |
|  |  |  |  |  |  |
| **Key Findings:** | | | | | |
| **Monitoring**: Collection of Urine Drug Samples was more frequent in PPCPM appointments, 98% versus 89% of psychiatrist-only appointments. | | | | | |

| **Year,**  **Author,**  **Country** | **Population** | **Duration** | **Inclusion Criteria** | **Exclusion Criteria** | **Participant Diagnoses, mean age (SD) years, % male overall** |
| --- | --- | --- | --- | --- | --- |
| 2021,  Mattle,  USA | Total Sample Size =150  Intervention (Multidisciplinary Practice) = 75  Control (Physician-only practice) = 75  Withdrawals = 8 | 5 years  and  8.5 months | Adults ≥18 years Diagnosis of opioid use disorder (OUD) Diagnostic and Statistical Manual of Mental Disorders (DSM)-IV or DSM-5 Initiated buprenorphine treatment between June 1, 2012, and Feb 13, 2018 In treatment for at least 28 days | 1.Pregnancy 2. Incarcerated or in custody of the criminal justice system | OUD,  37(11) (Intervention)  33(9.4) (Control)  Male %: 53 |
| **Aim and Pharmacist's Role:** | | | | | |
| To evaluate patient outcomes in a multidisciplinary outpatient buprenorphine program that includes clinical pharmacists, compared with a physician-only practice  Pharmacists provided (i) medication counselling, (ii) interpretation of toxicology screens, (iii) care coordination, (iv) recommendations for treatment adjustments, (v) participated in follow-up visits. | | | | | |
|  |  |  |  |  |  |
|  |  |  |  |  |  |
| **Key Findings:** | | | | | |
| **Medication Adherence:** Significantly more buprenorphine nonadherence was identified at the physician-only practice (p < 0.01). **Patient Reported**: Similar rates of engagement with counselling (p = 1) were observed in both groups. | | | | | |

| **Year,**  **Author,**  **Country** | **Population** | **Duration** | **Inclusion Criteria** | **Exclusion Criteria** | **Participant Diagnoses, mean age (SD) years, % male overall** |
| --- | --- | --- | --- | --- | --- |
| 2021,  Pohl,  USA | Total = 443 appointments  Intervention (Pharmacist-run appointments) = 381  Control (Psychiatrist-run appointments) = 62  Withdrawals = n/r | 2 years | Aged 18 or older Attention Deficit Hyperactivity Disorder (ADHD) diagnosis Completed a medication evaluation or medication follow-up visit from July 1, 2016 to June 30, 2019 | Another visit type, or  was a non-ADHD clinic provider visit,  or if the visit note was classified | ADHD,  IG: n/r.CG: n/r, n/r |
| **Description of Pharmacist's Role:** | | | | | |
| To measure the growth of an ADHD clinic in a college health center after the integration of clinical pharmacists and to evaluate provider adherence to clinic policies and procedures before and after pharmacist integration.  Pharmacists conducted collaborative initial visits with psychiatrists and independently provided follow-up appointments for patients with ADHD. The pharmacist integration aimed to supplement the psychiatrist's work by managing medication refills, monitoring for side effects, and providing counselling to patients about their ADHD treatment. | | | | | |
|  |  |  |  |  |  |
|  |  |  |  |  |  |
| **Key Findings:** | | | | | |
| **Monitoring:** Compared with psychiatrist-run appointments, pharmacist-run appointments were more adherent to monitoring blood pressure (11% vs. 77%, p < 0.001) and heart rate (6% vs. 75%, p < 0.001). | | | | | |

| **Year,**  **Author,**  **Country** | **Population** | **Duration** | **Inclusion Criteria** | **Exclusion Criteria** | **Participant Diagnoses, mean age (SD) years, % male overall** | |
| --- | --- | --- | --- | --- | --- | --- |
| 2020,  Salazar-Ospina, Colombia  **Follow up study from Salazar-Ospina 2017* | Total = 92  IG (Pharmaceutical Care (PC)) = 43  CG (Usual Care) = 49  Withdrawals = 0 | 1 year | Male or female patients between 18 and 65 years of age, with a diagnosis of BD-I according to the DSM-IV,  who participated in the EMDADER-TAB study and completed 12 months of follow-up | n/r | BD-I IG: 45.6 (10.1). CG: 47.4 (1.9), 43.5 | |
| **Aim and Pharmacist's Role:** | | | | | |  |
| To determine whether the effect of Pharmaceutical Care (PC) measured by the decrease in psychiatric hospitalizations and emergency service consultations is maintained one year after pharmacist intervention ceases  Patients in the IG received usual care, verbal and written counselling and PC for 1 year from a specially trained pharmacist using the Dader Method. The IG received a weekly telephone call from the pharmacist until the end of the study. During these calls, the pharmacist did the following: (a) carried out a clinical evaluation assessing changes in mood, behaviour, pattern of regular eating and sleeping, language, and thought; (b) emphasized the importance of patient education and the recognition and management of prodromal symptoms; (c) explained the proper use of bipolar drugs; (d) promoted therapeutic adherence; and (e) promoted healthy eating and lifestyle habits. After collecting the patient information, the pharmacist completed the assessment form and worked with the medical team, patient, and family/caregiver regarding any necessary interventions. | | | | | |  |
|  |  |  |  |  |  |  |
|  |  |  |  |  |  |  |
| **Key Findings:** | | | | | |  |
| **Hospitalisations**: One year after pharmacist intervention ceased, there were no significant differences between the groups in psychiatric hospitalisations (p = 0.261). There were 14 emergency service consultations for the IG versus 24 for the CG (p = 0.212). | | | | | |  |

| **Year,**  **Author,**  **Country** | **Population** | **Duration** | **Inclusion Criteria** | **Exclusion Criteria** | **Participant Diagnoses, mean age (SD) years, % male overall** |
| --- | --- | --- | --- | --- | --- |
| 2018, Sathien-  luckana, Thailand | Total Sample Size= 41  IG = 13  CG = 17  Withdrawals = 11 | 12 weeks | 1) aged between 18 and 50 years; 2) had been diagnosed with schizophrenia according to the DSM-IV; 3) had received stable agent and dosage regimen of antipsychotics for at least 2 months, 4) had mild to moderate symptoms of schizophrenia as determined by the Brief Psychiatric Rating Scale of 32–54; and 5) had schizophrenia after first diagnosis for a duration of less than 10 years. | Mental retardation, delirium, or had communication impairment, which affected ability to perform cognitive function tests. | Schizophrenia,  IG: 38.31 (4.96) and CG: 36.06 (9.88), 37 |
| **Aim and Pharmacist's Role:** | | | | | |
| To evaluate the impact of pharmacist intervention on cognitive outcomes in patients with schizophrenia by focusing on anticholinergic discontinuation  The pharmacist assessed symptom severity, anticholinergic discontinuation and cognitive functions in schizophrenia, medication history review, medication adherence, and identification of Drug Related Problems. In the IG, a pharmacist provided pharmaceutical care to all patients. These strategies included the following. 1) Avoiding or discontinuing medications that interfere with cognitive functions, especially the use of anticholinergic drugs in patients who had no extrapyramidal side effects (EPS) from antipsychotics for at least 4 weeks and no history of severe EPS. 2) Suggesting the addition of antidepressants or substitute second-generation antipsychotics (SGAs) for first-generation antipsychotics (FGAs) in patients who still exhibited predominant negative symptoms. 3) Considering the substitution of SGAs for FGAs in patients identified as having cognitive impairment based on cognitive function tests and with stable psychotic symptoms. 4) Decreasing the dose of antipsychotics in patients with EPS but whose other psychotic symptoms were stable. | | | | | |
|  |  |  |  |  |  |
|  |  |  |  |  |  |
| **Key Findings:** | | | | | |
| **Treatment Related**: DRPs were reduced by 85.19% in the pharmacist intervention group and by 9.76% in the usual care group. **Symptom Related**: Executive function perseverative errors within the pharmacist intervention group improved significantly from baseline (p = 0.003). Brief Psychiatry Rating Scale scores were significantly lower in the pharmacist intervention group than the usual care group (36.08 (±3.62) to 24.69 (±2.72) and 38.29 (±6.38) to 36.35 (±7.71), respectively; p < 0.001) | | | | | |

| **Year,**  **Author,**  **Country** | **Population** | **Duration** | **Inclusion Criteria** | **Exclusion Criteria** | **Participant Diagnoses, mean age (SD) years, % male overall** |
| --- | --- | --- | --- | --- | --- |
| 2018,  Lindell,  USA | Total Sample Size = 217  Intervention (care from psychiatrist and clinical pharmacist) = 100  Control (care solely from psychiatrist) = 117  Withdrawals = 0 | 23 months | Patients with depression and/or anxiety who had an appointment within the outpatient psychiatric clinic between January 2014 and November 2015 Age ≥ 18 years Diagnosis of depression and/or anxiety (ICD-9/10 coded) Underwent a psychotropic medication change (e.g., dose titration, switch) Had ≥1 return visit to the clinic | n/r | Depression, Anxiety, Other IG: 42 (n/r) CG:40 (n/r), 32 |
| **Aim and Pharmacist's Role:** | | | | | |
| To evaluate the difference in Patient Health Questionnaire (PHQ)-9 and/or GAD Questionnaire scores between the pharmacist-led and usual care groups.  Pharmacists participated in team meetings, conducted follow-up phone calls with up to 6 patients. Pharmacists provided telephonic follow-up care (~10–30 mins), including medication adherence assessment, side effect and tolerability monitoring, education on new medicines or dose changes, mood and suicidality check-ins, documentation of all contacts, recommendations to prescribers and in-clinic support. | | | | | |
|  |  |  |  |  |  |
|  |  |  |  |  |  |
| **Key Findings:** | | | | | |
| **Medication Adherence**: Patient self-reported adherence found a higher adherence rate in the intervention group (p < 0.0001) **Symptom Related**: No significant difference was found in PHQ-9 (p = 0.87) or GAD (p = 0.75) scores between groups. | | | | | |

| **Year,**  **Author,**  **Country** | **Population** | **Duration** | **Inclusion Criteria** | **Exclusion Criteria** | **Participant Diagnoses, mean age (SD) years, % male overall** |
| --- | --- | --- | --- | --- | --- |
| 2017,  Mishra,  India | Total Sample Size = 75  Intervention (Pharmacist and Psychiatrist) = 38  Control (Psychiatrist Only) = 35  Withdrawals = 2 | 3 months | Age: ≥ 18 years. Diagnosis: Diagnosed with Bipolar Affective Disorder (BPAD). Literate: Patients who were literate. | Comorbidities: BPAD patients with other comorbidities were excluded from the study. | BPAD, IG: 34.71 (10.65)  CG: 33.71 (11.17), 56 |
| **Aim and Pharmacist's Role:** | | | | | |
| To assess the impact of pharmacist–psychiatrist collaborative patient education on medication adherence and quality of life (QOL) of BPAD patients.    Patient information leaflets (PILs) specific to BPAD were developed by the investigator pharmacists to educate the patients with relevant disease conditions. Patient education provided by the pharmacist included awareness of the medications prescribed, disease, importance of adherence to medications and impact on overall QOL. Leaflets were used during patient education. IG patients were provided with patient education session during each follow-up. | | | | | |
|  |  |  |  |  |  |
|  |  |  |  |  |  |
| **Key Findings:** | | | | | |
| **Medication Adherence**: Improvement in medication adherence in the IG as measured by the MARS was found to be 2.06 (± 0.15) (p < 0.001) **Symptom Related**: Mean improvement in QOL, as measured by the WHO-BREF SCALE, of the test and control groups was found to be 13.8 (± 10.5) (p < 0.05) | | | | | |

| **Year,**  **Author,**  **Country** | **Population** | **Duration** | **Inclusion Criteria** | **Exclusion Criteria** | **Participant Diagnoses, mean age (SD) years, % male overall** |
| --- | --- | --- | --- | --- | --- |
| 2017, Mishra, India | Total Sample Size = 26  Intervention (Pharmacist and Psychiatrist) = 13  Control (Psychiatrist Only) = 10  Withdrawals = 3 | 3 months | Adults ≥18 years diagnosed with schizophrenia Literate patients. Patients receiving treatment for schizophrenia at the outpatient psychiatry department. | Patients with schizophrenia and co-morbidities treated in other departments. Non-literate patients. | Schizophrenia, n/r, 34.78 |
| **Aim and Pharmacist's Role:** | | | | | |
| To assess the impact of pharmacist–psychiatrist collaborative patient education on medication adherence and quality of life (QOL) of Bipolar Affective Disorder (BPAD) patients  Patient education was provided only to the intervention group by the Pharmacist (in addition to the usual care by psychiatrist) | | | | | |
|  |  |  |  |  |  |
| **Key Findings:** | | | | | |
| **Medication Adherence**: Both groups showed an increase in medication adherence as measured by the Medication Adherence Rating Scale (MARS). The intervention group had a larger and more statistically significant increase (p = 0.003) **Symptom Related**: Assessment of patient’s QOL, using the WHOQOL-BREF Scale between groups showed a mean (SD) improvement of 8.05(5.32) in QOL of the intervention group over the control group (p<0.001) | | | | | |

| **Year,**  **Author,**  **Country** | | **Population** | **Duration** | **Inclusion Criteria** | | **Exclusion Criteria** | | **Participant Diagnoses, mean age (SD) years, % male overall** | |  |
| --- | --- | --- | --- | --- | --- | --- | --- | --- | --- | --- |
| 2017,  Salazar-Ospina, Columbia | | Total Sample Size = 92  Intervention (Pharmaceutical Intervention) = 38  Control (Usual Care) = 43  Withdrawals =11 | 1 year | Patients diagnosed with Bipolar Disorder 1 (BD-I)  aged between 18 and 65 years and  discharged or referred from the outpatient service of the clinic. | | First manic episode, schizoaffective disorder, BD-II, cyclothymia, or other bipolar spectrum disorders; personality disorders and sociopathic disorder.  Epilepsy; HIV; or chronic decompensated disease.  Unable to comply with the protocol requirements because of severe alcohol and drug use.  Pregnancy or breastfeeding.  Intellectual disability, presence of any cognitive impairment or illiteracy. | | BD-I,   IG: 41.9 (9.9)   CG: 43.7 (11.8), 44 | |  |
| **Aim and Pharmacist's Role:** | | | | | | | | | |  |
| To assess the effectiveness of a pharmaceutical intervention using the Dader Method on patients with BD-I, measured by the decrease in the number of hospitalizations, emergency service consultations, and unscheduled outpatient visits from baseline through 1 year of follow-up.  Patients assigned to the IG received usual care and pharmaceutical care for 1 year. During the year of follow-up, the IG received a weekly telephone call from the pharmacist until the end of the study. During these calls, the pharmacist (a) carried out a clinical evaluation assessing changes in mood, behaviour, pattern of regular eating and sleeping, language, and thought; (b) emphasized the importance of patient education and the recognition and management of prodromal symptoms; (c) explained the proper use of bipolar drugs; (d) promoted therapeutic adherence; and (e) promoted healthy eating and lifestyle habits. The pharmacist then completed the assessment form and worked with the medical team, patient, and family/caregiver regarding any necessary interventions. | | | | | | | | | |  |
|  |  |  |  |  |  |  |  |  |  |  |
|  |  |  |  |  |  |  |  |  |  |  |
| **Key Findings:** | | | | | | | | | |  |
| **Hospitalisations**: The risk of hospitalisations and emergency service consultations was higher for the control group than for the intervention group (Hazard Ratio (HR) = 9.03, p = 0.042; HR = 3.38, p = 0.034, respectively) | | | | | | | | | |  |
| **Year,**  **Author,**  **Country** | **Population** | | **Duration** | | **Inclusion Criteria** | | **Exclusion Criteria** | | **Participant Diagnoses, mean age (SD) years, % male overall** | |
| 2017,  Singh,  India | Total Sample Size = 286  Intervention (Pharmacist Intervention) = 134  Control (Usual Care) = 132  Withdrawals = 20 | | 9 months | | Patients with BPAD, aged 18 to 65 years, who were discharged from the Outpatient Department (OPD). | | Patients with the history of epilepsy, schizophrenia, obsessive-compulsive disorder, alcohol-induced psychosis, mental retardation, pregnant and lactating women | | BAD, IG: 38.34 (12.91), CG: 36.6 (11.36), 62 | |
| **Aim and Pharmacist's Role:** | | | | | | | | | | |
| To compare and assess the impact of pharmaceutical care with the usual care for Patients with BAD.  The pharmacist provided the IG patient population with medication-related education, psycho-education, lifestyle modification education along with patient information leaflets and booklets. | | | | | | | | | | |
|  |  |  |  |  |  |  |  |  |  |  |
|  |  |  |  |  |  |  |  |  |  |  |
| **Key Findings:** | | | | | | | | | | |
| **Symptom Related**: In the IG, significant improvement in quality of life, as measured by the WHO-BREF scale, was observed during the study period (p < 0.001) | | | | | | | | | | |

| **Year,**  **Author,**  **Country** | **Population** | **Duration** | **Inclusion Criteria** | **Exclusion Criteria** | **Participant Diagnoses, mean age (SD) years, % male overall** |
| --- | --- | --- | --- | --- | --- |
| 2015,  Aljumah,  Saudi Arabia | Total Sample Size = 239 Intervention = 110 Control = 110 Withdrawals = 19 | 6 months | Aged 18 to 60 years; Newly diagnosed with a Major Depressive Disorder (MDD), according to the criteria of the DSM-IV; No history of psychosis or bipolar disorders; No drug or dependency history; No cognitive impairment that may hinder the assessment | No response at any level to the antidepressant within 8 weeks of recruitment. | MDD, not reported (n/r), 45 |
| **Aim and Pharmacist's Role:** | | | | | |
| To evaluate the effectiveness of shared decision making (SDM) - based pharmacist intervention for improving adherence and patient outcomes, compared with usual care in patients diagnosed with MDD.  The intervention focused on enhancing patients’ involvement in decision making by assessing their beliefs and knowledge about antidepressants. The average duration of the first SDM session (baseline) was 15 min, and the second session (final session) lasted 10 min (at 3-month follow-up) | | | | | |
|  |  |  |  |  |  |
|  |  |  |  |  |  |
| **Key Findings:** | | | | | |
| **Patient Reported:** Patient Satisfaction with treatment as measured by the Treatment Satisfaction Questionnaire for Medication was significantly higher in the intervention group (p < 0.0001) **Medication Adherence:** Patients in the intervention group had significantly more favourable medication adherence as measured by the Morisky Medication Adherance Scale (MMAS) (p < 0.0001) **Symptom Related:** The groups did not differ in severity of depression as measured by the Montgomery–Åsberg Depression Rating Scale (p=0.971) | | | | | |

| **Year,**  **Author,**  **Country** | **Population** | **Duration** | **Inclusion Criteria** | **Exclusion Criteria** | **Participant Diagnoses, mean age (SD) years, % male overall** |
| --- | --- | --- | --- | --- | --- |
| 2014, Schneider-han,  USA | Total Sample Size=120  Intervention Group (IG) = 60    Control Group (CG)=60    Withdrawals(after)=26 | 12 months | 1. Current Antispychotic Therapy  2. English Speaking  3. At least 18 years old  4. Competent to understand and make medical choices independently | 1. Patients previously seen by a comprehensive medication management pharmacist. | n/r, 42.9 (11.3) years, 40.8% |
| **Aim and Pharmacist's Role:** | | | | | |
| To determine the percentage of subjects taking antipsychotics who met criteria for metabolic ysndrome at baseline using point of care testing (POCT) and to evaluate the effectiveness of the provision by pharmacist comprehensive medication management regarding their ability to reduce the mean difference in the number of metabolic syndrome risk parameters based on point of care test results.  Provide Comprehensive Medication Management services (description not provided). Pharmacist used own judgement for scheduling follow-up vsit frequencies | | | | | |
|  |  |  |  |  |  |
|  |  |  |  |  |  |
| **Key Findings:** | | | | | |
| **Symptom related:** Between group differences in adjusted mean number of metabolic syndrome parameters at six months (p=0.24) and 12 months (p=0.99) were not statistically significant | | | | | |

| **Year,**  **Author,**  **Country** | **Population** | **Duration** | **Inclusion Criteria** | **Exclusion Criteria** | **Participant Diagnoses, mean age (SD) years, % male overall** |
| --- | --- | --- | --- | --- | --- |
| 2013,  Alves,  Brazil | Total = 58  IG = 26  CG = 22  Withdrawals = 10 | 3 months | Female gender Aged 18-65 years. A diagnosis of depression at the initial stage of treatment (first treatment, no previous antidepressant), or who were prescribed a new antidepressant | Insurmountable difficulties in scheduling visits, Becks Depression Inventory (BDI) <11 points, dependence on illicit drugs, schizophrenia diagnosis, or the presence of cognitive impairment. | Depression, IG: 40.8 (12.2),CG: 44.2 (13.9), 0 |
| **Aim and Pharmacist's Role:** | | | | | |
| To assess the effectiveness of Pharmaceutical care via Pharmacotherapy follow-up according to the Dáder Method in female patients diagnosed with depression.  The patients in the IG received pharmacist visits approximately every 30 days. They were given oral and written information about their treatment and educational lectures about disease and treatment. If problems related to pharmacotherapy were identified, the patients were informed and pharmaceutical intervention was performed between the pharmacist and patient or between the pharmacist, patient, and doctor. All interventions were recorded and included strategies to improve compliance with treatment, orientation regarding the disease and the patient’s medications, dose adjustment, substitutions of antidepressants, and the addition of medications. | | | | | |
|  |  |  |  |  |  |
|  |  |  |  |  |  |
| **Key Findings:** | | | | | |
| **Symptom Related:** A comparison of CG and IG showed a statistically significant difference between groups, with a median reduction in BDI score (Δ) of 2.5 points in the CG and 13.5 points in the IG (p = 0.0275). | | | | | |

| **Year,**  **Author,**  **Country** | **Population** | **Duration** | **Inclusion Criteria** | **Exclusion Criteria** | **Participant Diagnoses, mean age (SD) years, % male overall** |
| --- | --- | --- | --- | --- | --- |
| 2008,  Al-Saffar,  Kuwait | Total Sample Size = 150  Intervention Group (IG) (Leaflet) = 50  IG (Counselling) = 50  Control Group (CG) = 50  Withdrawals(after) = 71 | 6-8 weeks | New patients aged 18 or over who had been diagnosed with unipolar depression in accordance with the International Classification of Diseases 10^th^ edition (ICD-10) mood disorders criteria and who had been prescribed a Tricyclic Antidepressants (TCA) or Selective Serotonin Reuptake Inhibitor (SSRI) antidepressant. | 1. Patients with psychotic disorders, mental retardation, deafness, addiction or organic brain diseases. 2.Patients who were prescribed psychiatric medications other than TCAs and SSRIs, as well as those receiving a combination of TCAs and SSRIs antidepressants and  3. Those considered to be at a significant risk of suicide | Depression,   34 (9.5), 70 |
| **Aim and Pharmacist's Role:** | | | | | |
| To assess patients’ opinion toward receiving written or specialized verbal pharmacists’ interventions and to determine the effect of these interventions on patients’ medication knowledge.  Pharmacist counselling sessions were carried out in a private room and were between 10 and 15 min in length. The sessions were intended to help patients (i) understand the nature of their depressive illness and (ii) to reinforce that taking medications in the way they were prescribed would be of benefit to them. | | | | | |
|  |  |  |  |  |  |
|  |  |  |  |  |  |
|  |  |  |  |  |  |
| **Key Findings:** | | | | | |
| **Patient Reported:** Counselling was found to be significantly associated with a much higher recall of medicine name (Odds Ratio (OR) = 9.6, p =0.01), how to manage missed doses (OR = 8.9, p = 0.007), and correct use of medication (OR = 31.3, P < 0.001). Leaflet use was less strongly associated than counselling and was statistically significant for recall regarding correct use of medication (OR = 8.4, p = 0.009). | | | | | |
|  |  |  |  |  |  |

| **Year,**  **Author,**  **Country** | **Population** | **Duration** | **Inclusion Criteria** | **Exclusion Criteria** | **Participant Diagnoses, mean age (SD) years, % male overall** |
| --- | --- | --- | --- | --- | --- |
| 2006, Fridman, Argentina | Total Sample Size = 75  Group A (IG-Year 1, CG-Year 2) =34  Group B (CG-Year 1, IG-Year 2) = 35  Withdrawals = 6 | 2 years | Outpatients with a DSM-IV diagnosis of psychosis. Selected by a group of 17 psychiatrists participating in the study. Patients (or family members/dependents) who gave informed consent. | Pregnant women. Children. Patients who could not be followed up for some reason. Patients who changed their treating physician and/or diagnosis during the study. | Psychosis,  n/r, 49 |
| **Aim and Pharmacist's Role:** | | | | | |
| To evaluate the implementation of a Pharmaceutical Care program for outpatients with psychiatric problems in the pharmacy office and to compare it with traditional community pharmacy practice  Pharmacists conducted pharmacotherapeutic follow-up using structured tools, including: Pharmacological anamnesis, Monitoring of drug-related problems, Identification of habit-related problems, Identification and resolution of treatment non-specific factors. Collaborating with psychiatrists to: Resolve safety, adherence, and effectiveness issues, Educate patients and families about treatment. Providing individualized education to patients and caregivers on medication use and healthy habits. Supporting treatment adherence through regular follow-up and documentation. | | | | | |
|  |  |  |  |  |  |
|  |  |  |  |  |  |
| **Key Findings:** | | | | | |
| **Treatment Related**: Drug Related problems reduced significantly in both groups during the intervention period (Group A: 46.65% decrease, Group B: 52.63% decrease) | | | | | |

| **Year,**  **Author,**  **Country** | **Population** | **Duration** | **Inclusion Criteria** | **Exclusion Criteria** | **Participant Diagnoses, mean age (SD) years, % male overall** |
| --- | --- | --- | --- | --- | --- |
| 1993, Dorevitch, Israel | Total Sample Size = 14  Intervention (pharmacist involvement) = 14  Control (Usual Care prior to pharmacist involvement) = 14  Withdrawals = 0 | 10 years | Patients diagnosed with schizophrenia attending this out-patient clinic | n/r | Schizophrenia 39 (n/r), 71 |
| **Aim and Pharmacist's Role:** | | | | | |
| To describe the experience of a psychiatric clinical pharmacist acting in a primary care function under the supervision of a psychiatrist to monitor medication for chronic schizophrenic out-patients over a 10-year period.  The clinical pharmacist performed an informal mental status assessment, and monitored the patient for extrapyramidal side effects, tardive dyskinesia, and other central and peripheral side-effects of psychotropic drugs.  Counselling was provided on compliance, dosage and administration questions, side-effects and major contraindications. A medication profile was maintained on all patients. After the interview, the clinical pharmacist made a progress note on the patient‘s chart. After each clinic a supervising session was conducted with the psychiatrist to review each patient‘s case and to have the psychiatrist co-sign the clinical pharmacist's progress notes. | | | | | |
|  |  |  |  |  |  |
|  |  |  |  |  |  |
| **Key Findings:** | | | | | |
| **Treatment Related:** No. of medication-related side-effects per patient was significantly less at the end of the study period (p = 0.0033) **Hospitalisations**: No. of days of hospitalisation for psychiatric reasons reduced from 684 days in the 10-year pre study period to 102 days in the 10-year study period. | | | | | |

| **Year,**  **Author,**  **Country** | **Population** | **Duration** | **Inclusion Criteria** | **Exclusion Criteria** | **Participant Diagnoses, mean age (SD) years, % male overall** |
| --- | --- | --- | --- | --- | --- |
| 1989,  Lobeck,  USA | The study was visit-based, not patient-based, so the sample size is reported as: Pre-intervention (6 months): 4,734 clinic visits Intervention period (3 months): 2,662 clinic visits | 9 months | Not explicitly stated.  Implied inclusion: All patients attending the outpatient mental health clinic during the pre- and post-intervention periods whose visits generated prescriptions that were recorded in the pharmacy system. | n/r | n/r   n/r   n/r |
| **Aim and Pharmacist's Role:** | | | | | |
| To evaluate the economic impact of implementing a clinical pharmacy service in an outpatient mental health clinic, by comparing pharmacy-related costs and prescribing patterns before and after the intervention.  All charts were reviewed by the clinical pharmacist the day before patients’ scheduled clinic visits. The pharmacist wrote recommendations about the patient’s psychotropic medication treatment and attached them to the chart for the treating psychiatrist’s consideration. The pharmacist educated patients about medication, provided pharmacokinetic dosing services, monitored for adverse effects, provided drug information services to the clinicians, and performed drug use evaluations. | | | | | |
|  |  |  |  |  |  |
|  |  |  |  |  |  |
| **Key Findings:** | | | | | |
| **Cost**: Total Cost per Prescription (Drug + Personnel) decreased by 14.3% by the end of the intervention period. The Drug cost per prescription decreased by 34.6% across the same period. | | | | | |
